# Wild Edible Fruits: A Structured Narrative Review on Bioactive Composition and Bioactivity

**DOI:** 10.3390/foods15061106

**Published:** 2026-03-22

**Authors:** Carlos Díaz-Romero, Jesús Heras-Roger, Miguel Ángel Rincón-Cervera, José Luis Guil-Guerrero

**Affiliations:** 1 Department of Chemical Engineering and Pharmaceutical Technology, University of La Laguna, 38203 Santa Cruz de Tenerife, Spain; cdiaz@ull.edu.es (C.D.-R.); jherasro@ull.edu.es (J.H.-R.); 2 Cátedra de Agroturismo y Enoturismo de Canarias, Instituto Canario de Calidad Agroalimentaria (ICCA-ULL), 38001 Santa Cruz de Tenerife, Spain; 3 Institute of Nutrition and Food Technology, University of Chile, Santiago 7830490, Chile; marincer@inta.uchile.cl; 4 Department of Agronomy, Food Technology Area, University of Almería, 04120 Almería, Spain

**Keywords:** wild edible fruits, antioxidant activity, bioactive compounds, phytochemicals, ethnomedicine, nutraceuticals, functional foods, food security, sustainable use, biodiversity conservation

## Abstract

Wild edible fruits (WEFs) represent an important yet underutilised component of biodiversity-based nutrition and functional food research. This structured narrative review critically synthesises current evidence on the phytochemical composition and nutritional relevance, biological activities, and sustainability dimensions of WEFs, with emphasis on fruit pulp as the primary edible tissue. A systematic search strategy following PRISMA-based principles was applied to enhance methodological transparency; however, due to high heterogeneity in species, analytical methods, and outcome measures, quantitative meta-analysis was not feasible. The review integrates compositional data (phenolics, carotenoids, tocopherols, sterols, vitamin C, and minerals) with reported bioactivities, while explicitly distinguishing between in vitro assays, in vivo studies, and limited clinical evidence. Particular attention is given to analytical variability, bioavailability constraints, dose–response relationships, and translational limitations that affect the interpretation of antioxidant and other health-related claims. Beyond bioactivity, the manuscript contextualises WEFs within socio-economic, conservation, and sustainable food system frameworks. By combining chemical characterisation, evidence hierarchy, and sustainability analysis, this review provides a critical and multidisciplinary perspective that advances understanding of WEFs and identifies priorities for future research, including standardised methodologies and well-designed human intervention trials.

## 1. Introduction

Wild edible fruits (WEFs) are naturally occurring, uncultivated plant resources that have long played a fundamental role in the diets, health practices, and cultural traditions of indigenous and rural communities worldwide. Beyond their dietary importance, WEFs contribute significantly to biodiversity conservation, food security, and the socioeconomic resilience of vulnerable populations, particularly in developing regions [1,2]. Ethnobotanical surveys have documented a remarkable diversity of WEFs across continents, for instance, including 43 species in the Kolli Hills of India [1], 35 species in the Lesser Himalayas of Pakistan [3], and 69 species in the Garhwal region of Uttarakhand, India [2], underscoring their widespread availability and cultural relevance.

From a nutritional perspective, WEFs constitute valuable sources of both macro- and micronutrients essential for human nutrition, providing a diverse range of dietary components that contribute to balanced and nutrient-dense diets. Their carbohydrate and dietary fibre contents are often comparable to those of widely consumed cultivated fruits; for example, *Mimusops elengi* (18.1%) and *Ziziphus rugosa* (20.7%) provide carbohydrate levels similar to those of mango and pomegranate [4]. Wild fruits traditionally used by the Orang Asli communities in Malaysia are particularly rich in fibre and protein [5]. Protein content in species such as *Bridelia tomentosa* (3.1%) and *Carissa spinarum* (3.6%) approaches that of guava and banana, while *Tamarindus indica* stands out for its high protein concentration (≈60%) [6]. Although most wild fruits are characteristically low in fat, certain species—such as *Neolitsea pallens*, with lipid contents reaching 70.4%—constitute notable exceptions and may serve as valuable energy-dense foods [7]. In addition to macronutrients, WEFs constitute important sources of micronutrients that are frequently deficient in local diets. For example, several species consumed in northeastern Madhya Pradesh (India) have been reported to contain notable concentrations of essential minerals and vitamins [8]. Many species contain high levels of vitamin C, frequently exceeding those found in cultivated fruits. For instance, *Solanum torvum* and *Terminalia citrina* display superior ascorbic acid concentrations [4]. Likewise, many studies report high levels of essential minerals, e.g., calcium, iron, and zinc. Wild orange is notable for its sodium, potassium, and magnesium content [9], whereas *Tamarindus indica* provides substantial amounts of calcium and zinc [6]. These nutritional attributes highlight the potential of WEFs to complement conventional diets, prevent micronutrient deficiencies, and promote overall health. They could be used to design strategies aimed at reducing malnutrition and diet-related diseases [1,8]. In addition to their nutritional value, WEFs are deeply embedded in traditional knowledge systems and ethnomedicinal practices. Across different regions, fruits are consumed fresh or processed into powders, decoctions, pastes, or preserves, depending on cultural preferences and medicinal uses. In the Lesser Himalayas of Pakistan, for instance, WEFs are employed in multiple preparations to address a range of ailments [3]. However, patterns of consumption and knowledge transmission are changing. In the Garhwal Himalaya, generational shifts, rural–urban migration, and declining interest among younger populations have contributed to reduced use and loss of traditional knowledge associated with WEFs [2].

The conservation of WEFs is therefore essential not only for maintaining plant biodiversity but also for safeguarding cultural heritage and supporting sustainable development. Community-driven conservation approaches have shown promising results in several regions. In Benin, for example, local beliefs, cultural norms, and religious practices play a central role in protecting species such as *Strychnos spinosa* [10]. Similarly, in the Indian Himalayan Region, the preservation of traditional ecological knowledge and the promotion of sustainable harvesting practices are increasingly recognised as priorities for the long-term viability of WEF resources [11].

Despite their importance, WEFs face multiple and interrelated challenges, including habitat loss, agricultural expansion, overexploitation, erosion of traditional knowledge, and changing dietary preferences. These pressures threaten both species diversity and the continuity of associated cultural practices [1,10,12]. Addressing these challenges requires integrated conservation strategies that combine sustainable resource management, documentation of indigenous knowledge, and incorporation of WEFs into local and regional food systems. Such integration can enhance food security, diversify diets, and provide alternative livelihood opportunities, while simultaneously promoting biodiversity conservation [2,11].

Several comprehensive reviews have addressed WEFs from the perspectives of biodiversity conservation and nutritional potential [13,14], systematic mapping of multifunctional non-timber forest products [15], sustainability and health interfaces [16], or broader phytochemical and food-use syntheses [17]. However, these studies primarily emphasise species inventories, conservation frameworks, or general ethnobotanical and nutritional overviews. In addition, the present review differentiates itself by integrating detailed comparative analysis of fruit pulp phytochemical composition, critical evaluation of analytical standardisation and evidence hierarchy (in vitro vs. in vivo vs. clinical validation), and socio-economic and sustainability dimensions within a unified framework. Furthermore, although numerous regional reviews exist focusing on locally important wild fruits, their geographically restricted scope and heterogeneous analytical approaches limit cross-species comparison. By combining compositional rigor with translational and socio-economic analysis, this review aims to provide a more integrative and critical synthesis of the functional and sustainable potential of WEFs.

In this context, WEFs represent a largely underutilised resource with considerable potential for improving human nutrition, supporting traditional healthcare systems, and fostering sustainable development. A comprehensive synthesis of their diversity, bioactive composition, and biological activities is therefore essential to inform future research, conservation policies, and practical applications in nutrition and health. This review aims to synthesise current knowledge on the bioactive composition and biological activities of WEFs, with a particular focus on compounds present in the fruit pulp—the primary edible tissue—while excluding seeds, peels, and other non-pulp components unless otherwise specified.

## 2. Materials and Methods

A structured and systematic approach was applied to identify, screen, and synthesise published studies addressing the diversity, bioactive composition, and biological activities of WEFs. Although informed by PRISMA principles to enhance transparency and traceability of sources, this review does not constitute a formal systematic review, and no risk-of-bias assessment or meta-analysis was performed.

### 2.1. Literature Search Strategy

An extensive literature search was performed across major scientific databases, including PubMed, Scopus, ScienceDirect, SpringerLink, and Google Scholar. These platforms were selected to ensure broad coverage of peer-reviewed literature spanning food science, nutrition, ethnobotany, pharmacology, and environmental sciences.

A targeted keyword strategy was employed, combining terms related to wild edible fruits, phytochemical composition, and biological activities. Search strings included combinations such as “wild edible fruits AND bioactive compounds”, “wild fruits AND sterols”, “phenolics OR flavonoids AND wild fruits”, and “fatty acids OR lipids AND wild fruits”. Boolean operators (AND, OR) were systematically applied to refine and optimise the search results.

**Figure 1:**
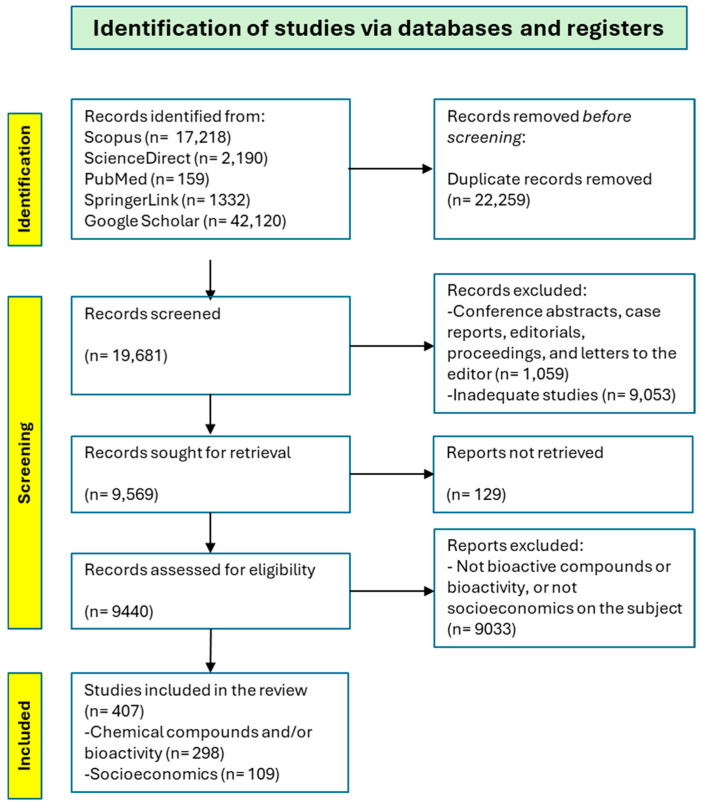
The research flow diagram conducted to perform this review.

In addition to database searches, backward citation tracking was conducted by screening the reference lists of key reviews and highly relevant primary studies. This approach enabled the identification of additional publications not captured through electronic searches. The overall study selection process is summarised in the study selection flowchart shown in Figure 1.

### 2.2. Eligibility Criteria and Study Selection

The eligibility criteria were defined using the PICO (Population, Intervention, Comparator, Outcome) framework [18], which is widely used in systematic reviews to structure research questions and guide study selection.

- Population: Species of WEFs.
- Intervention: Analysis of chemical composition and/or bioactivity.
- Comparator: Comparison of positive biological effects *versus* neutral or negative actions, where applicable.
- Outcome: Identification of bioactive compounds, biological activities, nutritional relevance, and potential applications of WEFs.

The PICO framework facilitated consistent and unbiased screening across databases and ensured that selected studies were directly relevant to the objectives of this review.

### 2.3. Inclusion and Exclusion Criteria

Only peer-reviewed articles published in English were considered eligible for inclusion. Studies were required to focus explicitly on WEFs and to report at least one of the following topics:

1. Detailed characterisation of bioactive compounds.
2. Results from bioactivity assays (e.g., antioxidant, antimicrobial, anti-inflammatory, or cytotoxic activities).
3. Nutritional, biomedical, or functional applications.
4. Ecological, ethnobotanical, or socioeconomic implications related to WEF use.
5. No strict publication date limits were imposed. However, priority was given to publishing studies from the 1980s onward, a period in which systematic chemical and biological analyses of WEFs became widespread. Previously published seminal studies were selectively included when they provided foundational data on the composition or traditional use of WEFs.

Exclusion criteria comprised non–peer-reviewed sources, studies lacking sufficient methodological detail, articles not focused on wild or underutilised fruit species, and publications addressing cultivated fruits without a clear link to wild relatives.

### 2.4. Data Extraction and Synthesis

Relevant data were extracted from eligible studies and organised into thematic categories, including species diversity, bioactive compound classes, analytical methods, biological activities, and nutritional or functional implications. Where available, quantitative data were synthesised from systematic reviews and meta-analyses to identify general trends and ranges of compound concentrations or bioactivities.

In addition, studies addressing sustainability, life-cycle assessments, and environmentally friendly extraction techniques were reviewed to contextualise the valorisation of WEFs within sustainable food and bioeconomy frameworks. This integrative approach allowed for a comprehensive evaluation of both the biological potential and the broader ecological and societal relevance of WEFs.

## 3. Diversity of Wild Edible Fruits

### 3.1. Regional Case Studies

WEFs exhibit remarkable regional diversity in terms of species composition, cultural relevance, and modes of utilisation. Across different geographical contexts, these fruits play multifunctional roles as sources of nutrition, traditional medicine, and household income, reflecting close interactions between local communities and their surrounding ecosystems.

In Northeast India, WEFs are deeply integrated into ethnomedicinal systems and are traditionally used to manage gastrointestinal disorders, respiratory ailments, cardiovascular conditions, and infectious diseases such as malaria [19]. Similarly, in Arunachal Pradesh, a biodiversity-rich region of the eastern Himalayas, wild fruits serve both therapeutic and dietary functions while also contributing to local livelihoods and cultural identity [20]. These practices highlight the dual nutritional and medicinal roles of WEFs within indigenous healthcare systems.

In southern Africa, WEFs form an essential component of traditional ecological knowledge and subsistence strategies. Their use reflects long-standing environmental knowledge, seasonal resource management, and adaptive survival skills in communal landscapes [21]. Beyond subsistence, WEFs also contribute to rural economies. In Central Kalimantan (Indonesia), species such as *Durio kutejensis* (Durian Pulu) represent important income-generating resources for households, despite persistent challenges related to land tenure insecurity, habitat conversion, and limited market access [22]. Comparable patterns are observed in East Aceh (Indonesia), where fruits such as *Mangifera odorata* (Kuini) and *Durio oxleyanus* provide both nutritional benefits and economic value to local communities [23].

Several case studies further illustrate the nutritional and bioactive potential of region-specific WEFs. In the Himalayan region (India), *Myrica esculenta* (bayberry) and *Rubus ellipticus* (yellow Himalayan raspberry) are widely consumed and valued for their high antioxidant capacity and nutrient density [24,25]. In the Amazon basin, cocona (*Solanum sessiliflorum*) is recognised as an important wild food resource due to its appreciable fibre, protein, and mineral content, contributing to dietary diversification in riverine and forest-dependent populations [26].

Despite their demonstrated importance, significant research gaps remain across regions. Many WEF species lack comprehensive documentation regarding their nutritional composition, bioactive profiles, traditional uses, and commercialisation potential [15,27]. In addition, socioeconomic and institutional barriers—including insecure land rights, weak value chains, and limited policy recognition—often constrain sustainable use and market integration. Addressing these challenges requires coordinated policy interventions that strengthen land tenure security, improve market access, and promote the integration of traditional ecological knowledge with modern scientific and agronomic practices [22,28].

### 3.2. Conservation Status and Threats

The conservation status of WEFs varies widely across regions, reflecting differences in ecological conditions, land-use dynamics, and the extent of scientific and institutional assessment. An overview of the conservation status, key threats, and management strategies for WEFs across selected regions is presented in Table 1. In many biodiversity-rich areas, a substantial proportion of WEF species remain unclassified or insufficiently evaluated, primarily due to limited data availability. For example, in Aceh Province (Indonesia), although several species have been categorised as Least Concern [29], more than half lack formal conservation status, highlighting significant gaps in biodiversity assessment and monitoring [23]. This situation underscores the urgent need for systematic inventories and conservation evaluations to support evidence-based management.

Mountain ecosystems, particularly the Himalayan region, are recognised as global biodiversity hotspots and harbour a wide diversity of wild fruit species that are essential for local food security and rural livelihoods. However, these ecosystems are highly vulnerable to environmental and anthropogenic pressures. In the Indian Himalayas, increasing attention has been directed toward scientific propagation, sustainable harvesting, and conservation-oriented agroforestry systems as strategies to ensure the long-term availability of WEFs [28,30]. In Arunachal Pradesh, the rapid decline of wild fruit populations has been linked to overexploitation and habitat disturbance, prompting calls for the promotion of sustainable agroforestry practices and supportive policy frameworks [20].

Similar conservation challenges have been documented in other regions. In China, wild fruit species belonging to the Annonaceae family face increasing pressure from unsustainable harvesting and habitat loss, exacerbated by limited public awareness and insufficient scientific documentation [29]. Across sub-Saharan Africa, WEFs are threatened by agricultural expansion, deforestation, overgrazing, selective logging, and land-use change, all of which compromise species regeneration and ecosystem resilience [31,32,33,34,35,36]. Urbanisation and charcoal production further intensify pressure on wild fruit resources in countries such as Tanzania [12].

**Table 1:** Conservation Status and Threats of Wild Edible Fruits.

Region	Conservation Status	Threats	Key Species	Conservation Strategies	Reference
Aceh Province (Indonesia)	35% Least Concern; 6% Vulnerable; 3% Near Threatened; 2% Low Risk; 2% Data Deficient; 52% No Data	Agricultural expansion; Over-exploitation; Land-use changes	*Mangifera foetida*; *M. odorata*; *Artocarpus integer*; *Ficus altissima*; *Syzygium cumini*	Community-based conservation; Domestication of WEFs	[23,37]
Paser, East Kalimantan (Indonesia)	Not specified	Agricultural expansion; Mining; Deforestation	*Baccaurea lanceolata*	In situ and ex situ conservation; Local community involvement	[22]
Ethiopia	Not specified	Habitat degradation; Agricultural expansion; Overgrazing; Selective logging; Deforestation	*Opuntia ficus-indica*; *Carissa edulis*; *Ficus vasta*	Community-based conservation; Awareness-raising; Sustainable Forest management	[31,32,33,34,35,36]
Garhwal Himalaya (India)	Vulnerable	Overharvesting; Habitat disturbances	*Paeonia emodi*	Agro-production techniques; Sustainable utilisation	[38]
Arunachal Pradesh (India)	Not specified	Rapid vanishing rate; Over-exploitation	Various species	Agroforestry systems; Policy interventions	[20]
Nepal	Not specified	Habitat destruction; Land-use change; Over-harvesting	Various species	Sustainable collection and trade; Community engagement	[39]
Tanzania	Not specified	Urbanisation; Agricultural expansion; Charcoal activities	*Vitex mombassae*; *Strychnos spinosa*; *Tamarindus indica*	Market promotion; Cultural importance awareness	[12]

Despite regional differences, the threats faced by WEFs share common characteristics. Agricultural intensification, habitat degradation, overharvesting, and weak regulatory frameworks are recurrent drivers of population decline. These pressures are often exacerbated by the erosion of traditional ecological knowledge and the limited awareness of the nutritional and economic value of WEFs [12,20,22,23,37,38]. Without targeted interventions, these factors collectively jeopardise both biodiversity conservation and the livelihoods that depend on WEFs.

In response, a range of conservation and management strategies has been implemented with varying degrees of success. Community-based conservation initiatives, sustainable harvesting guidelines, domestication and semi-domestication of priority species, and the integration of WEFs into agroforestry systems have emerged as effective approaches for balancing conservation and use [12,20,22,23,31,32,38,39]. These strategies not only support species conservation but also enhance local livelihoods, strengthen cultural resilience, and promote sustainable resource governance.

## 4. Bioactive Compound Profiles

WEFs are characterised by a rich and diverse phytochemical composition that underpins their nutritional value, health-promoting properties, and traditional medicinal uses. These fruits contain a broad spectrum of bioactive compounds, including phenolic acids and flavonoids, carotenoids and other pigments, tocols, sterols, vitamins, essential minerals, terpenoids, polysaccharides, dietary fibres, and fatty acids. The qualitative and quantitative composition of these compounds varies widely among species and is influenced by genetic background, environmental conditions, maturity stage, and post-harvest handling.

Many of the biological activities attributed to WEFs—such as antioxidant, anti-inflammatory, antimicrobial, cardioprotective, and neuroprotective effects—are closely linked to the presence and synergistic interactions of these bioactive constituents. Phenolic compounds and carotenoids, for example, play a central role in mitigating oxidative stress through free radical scavenging and redox regulation, whereas tocols and sterols contribute to lipid protection and cardiovascular health. Vitamins and essential minerals further enhance metabolic and immune functions, whereas terpenoids and essential oils are often responsible for antimicrobial activity and distinctive sensory properties.

The growing scientific interest in WEFs has been driven by advances in analytical techniques, including high-performance liquid chromatography, gas chromatography, mass spectrometry, and spectrophotometric assays, which have enabled detailed characterisation of phytochemical profiles and bioactivities. However, substantial variability in reported values persists due to differences in extraction methods, analytical protocols, and units of expression. Consequently, careful comparison and critical interpretation of data across studies is essential.

The following subsections synthesise current knowledge on the major classes of bioactive compounds identified in WEFs. Emphasis is placed on their chemical characteristics, occurrence in selected species, reported concentrations, and documented biological activities, providing a comprehensive framework for understanding the functional potential of WEFs in nutrition, health, and sustainable food systems.

### 4.1. Phenolic Compounds

Phenolic compounds are products of secondary metabolism from plants, and they are non-essential human nutrition constituents. These compounds are among the most abundant and biologically relevant phytochemicals in WEFs [40], contributing to colour, sensory quality, and health-promoting properties. They can be classified into the following major groups: phenolic acids, flavonoids, tannins, anthocyanins, stilbenes, and lignans with structures ranging from simple phenolic molecules to complex high-molecular-mass polymers [41], which are widely recognised for their ability to modulate oxidative stress, a key factor in the development of chronic diseases [38,42,43,44,45,46].

The phenolic compounds of grapes, such as resveratrol, among many others, are good antioxidants, which implies a decrease in the incidence of degenerative diseases [47]. Berries, especially members of several families, such as the Rosaceae family (strawberry, raspberry, or blackberry), and the Ericaceae family (blueberry and cranberry), belong to the best dietary sources of phenolic compounds (phenolic acids, flavonoids, such as anthocyanins and flavonols, and tannins) [48]. These compounds, either individually or in combination, are responsible for several health benefits of berries, such as the prevention of inflammatory disorders and protective effects against various cancers. Proanthocyanidins are phenolic compounds responsible for the violet colour of blueberries. These antioxidant compounds decrease the adhesion of *Escherichia coli* and other pathogenic bacteria to renal tubules, which improves the urinary infection symptoms [49].

As summarised in Table 2, total phenolic contents vary widely among WEF species, ranging from very low levels in *P. alkekengi* and *S. cordatum* (<0.3 mg/g fw) to high concentrations in *D. decandra*, *C. monogyna*, *I. malayana*, and *R. canina* (>100 mg/g dw in some cases). Fruits from the Rosaceae, Combretaceae, and Ebenaceae consistently emerge as phenolic-rich taxa, supporting their prominent roles in traditional diets and medicinal practices.

Qualitatively, WEFs contain diverse phenolic acids (e.g., gallic, chlorogenic, caffeic, ferulic) and flavonoids (e.g., catechin, quercetin, rutin), with anthocyanin-rich species such as *S. nigra*, *V. myrtillus*, *Rubus* spp., and wild *Prunus* species characterised by high levels of cyanidin derivatives (Table 2). These compounds are associated not only with strong antioxidant activity but also with anti-inflammatory, antimicrobial, antidiabetic, and anticancer effects [44,45,50].

Substantial variability in reported phenolic values reflects differences in species, geographic origin, and analytical methodology. As evidenced in Table 2, data are reported on both fresh- and dry-weight bases and obtained using different extraction solvents and analytical techniques, with polar solvents generally yielding higher phenolic recoveries [44,51]. This heterogeneity highlights the need for methodological harmonisation to improve comparability across studies.

**Table 2:** Phenolic content and profiles of the pulp of selected Wild Edible Fruits.

Family	Fruit	Total Phenolics (GAE) *	Main Phenolics	Reference
Adoxaceae	*Sambucus nigra* (Elderberry)	12.7 mg/g fw	Cyanidin-3-galactoside chloride	[52]
	*Viburnum foetens*	1.14 mg rutin Equivalent/g fw	Flavonoids, flavonols, phenolics	[53]
Arecaceae	*Euterpe edulis*	0.75–1.37 mg/g fw (HPLC)	Gallic and protocatechuic acids, epicatechin, and quercetin	[54]
Berberidaceae	*Berberis asiatica*	High levels (not specified)	Catechin, anthocyanins (cyanin, delphinidin)	[55]
	*Berberis crataeginea*	0.02–0.23 mg/g fw	Rutin trihydrate, *trans*-cinnamic acid	[56]
Combretaceae	*Terminalia chebula*	14.03 mg/g dw	Protocatechuic, vanillic, and ferulic acids	[57]
Cornaceae	*Cornus mas* (Cornelian cherry)	7.31–14.39 mg/g dw	Chlorogenic acid, caffeic acid, epicatechin, quercetin, cyanidin-3-O-glucoside, pelargonidin	[58]
Ebenaceae	*Diospyros decandra*	214.64 mg/g dw	Caffeic and syringic acids	[57]
Ericaceae	*Arbutus unedo*	7.73–16.21 mg/g fw	Gallic acid, cyanidin 3-glucoside	[59]
	*Vaccinium myrtillus* (Bilberry)	10.4 mg/g fw	Cyanidin-3-galactoside chloride	[52]
Irvingiaceae	*Irvingia malayana*	121.57 mg/g dw	Not specified	[60]
Muntingiaceae	*Muntingia calabura*	16.5 mg/g dw	Ferulic acid	[57]
Myricaceae	*Myrica esculenta*	9–15 mg/g fw	Catechin, anthocyanins (cyanin, delphinidin)	[55]
Myrtaceae	*Myrtus comunis*	0.38 mg/g fw	Gallic acid, catechin, quercetin, Isorhamnetin, Apigenin 7-glucoside	[61]
	*Syzygium cordatum*	0.02 mg/g fw	Flavonols, phenolic acids	[62]
Olacaceae	*Ximenia caffra*	12.05 mg/g fw	Flavonols, phenolic acids	[62]
Phyllanthaceae	*Antidesma velutinosum*	0.106 mg/g fw (HPLC)	Quercetin, caffeic, ferulic acids	[63]
Rhamnaceae	*Zizyphus lotus*	16.4 mg/g dw (HPLC)	Gallic, vanillic, *p*-hydroxybenzoic, chlorogenic acids	[64]
Rosaceae	*Crataegus monogyna*	101–153 mg/g dw	Epicatechin, hyperoside, chlorogenic acid, luteolin, quercetin, catechin, cyanidin	[65,66]
	*Prunus avium* (Wild Cherry)	8.8 mg/g fw	Cyanidin-3-galactoside chloride	[52]
	*Prunus species* (Wild cherry)	2.37–11.05 mg/g fw	Cyanidin-3-rutinoside, cyanidin-3-glucoside, quercetin derivatives	[67]
	*Rosa canina*	44.6–157 mg/g dw	Gallic and cinnamic acids, rutin, and quercetin	[68]
	*Rubus fruticosus* (Blackberry)	9.8 mg/g fw	Cyanidin-3-galactoside chloride	[52]
	*Rubus ulmifolius*	3.76–13.26 mg/g fw	Gallic acid, cyanidin 3-glucoside	[59]
Rutaceae	*Citrus daoxianensis*	43.46–45.38 mg/g dw	Vanillic, caffeic, *p*-cumaric, ferulic acids	[69]
	*Citrus poonensis*	36.54 mg/g dw	Vanillic, caffeic, *p*-cumaric, ferulic acids	[69]
	*Citrus reticulata*	29.38–51.14 mg/g dw	Vanillic, caffeic, *p*-cumaric acids	[69]
Solanaceae	*Physalis alkekengi*	0.01–0.24 mg/g fw	*p*-Coumaric acid, (+)-catechin	[56]

* GAE: Gallic Acid Equivalents.

### 4.2. Carotenoids and Pigments

**Figure 2:**
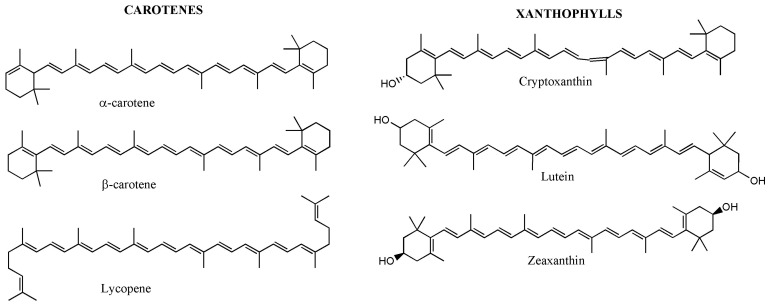
Chemical structure of the main carotenoids occurring in Wild Edible Fruits.

Carotenoids are lipid-soluble pigments responsible for the yellow, orange, and red colouration of many WEFs. They are structurally classified into carotenes (e.g., β-carotene, α-carotene, lycopene) and xanthophylls (e.g., lutein, zeaxanthin, violaxanthin) (Figure 2) and play key roles in human nutrition as provitamin A compounds, antioxidants, and regulators of immune and visual functions [70,71,72].

From a nutritional perspective, β-carotene is particularly important due to its conversion into retinol, which is essential for vision, immune competence, epithelial integrity, and growth. Despite the availability of carotenoid-rich foods, vitamin A deficiency remains prevalent in many regions, especially among children, highlighting the need for diversified and bioavailable dietary sources [73,74]. In this context, WEFs represent an underutilised yet nutritionally valuable resource.

The data summarised in Table 3 reveal substantial interspecific variability in carotenoid compounds and their contents among WEFs. Total carotenoid concentrations range from very low levels in species such as *L. ruthenicum* (2.4 mg/kg dw) to high levels in *E. umbellata* (1851 mg/kg dw), *H. thebaica* (1467 mg/kg dw), and *H. rhamnoides* (531–967 mg/kg dw). These species emerge as a particularly rich source of carotenoids with strong potential to contribute to vitamin A intake and antioxidant defence.

Several WEFs exhibit complex carotenoid profiles rather than dominance by a single compound. For example, *C. macranthus* contains zeaxanthin, violaxanthin, β-cryptoxanthin, and capsanthin derivatives, with higher concentrations in the aril than in the mesocarp, indicating tissue-specific accumulation [75]. Similarly, *C. monogyna* and *R. canina* contain diverse mixtures of β-carotene, cryptoxanthin’s, lutein, and lycopene, supporting their multifunctional antioxidant and nutritional roles [76,77].

Xanthophyll-rich species such as *A. unedo*, *P. torta*, and several *Eugenia* species are notable for their lutein and violaxanthin content, compounds associated with retinal protection and reduced risk of age-related macular degeneration [78,79]. In contrast, lycopene-rich fruits such as *E. umbellata* and *R. canina* are linked to enhanced singlet oxygen quenching and potential cardioprotective and anticancer effects [70,80].

Carotenoid bioavailability is strongly influenced by food matrix and processing conditions. While the presence of dietary lipids enhances absorption, excessive thermal processing and prolonged cooking can promote oxidative degradation and isomerisation, reducing nutritional value [71,80]. These factors are particularly relevant in traditional food systems, where preparation practices may inadvertently limit carotenoid retention.

**Table 3:** Carotenoid composition of the pulp of selected Wild Edible Fruits.

Family	Species	Carotenoids	Total Carotenoids mg/kg	Reference
Apocynaceae	*Carissa carandas*	Zeaxanthin,	559 dw	[81]
Arecaceae	*Hyphaene thebaica*	β-carotene, lutein	1467 dw	[82]
Cucurbitaceae	*Cionosicyos macranthus*	Zeaxanthin, Violaxanthin, β-cryptoxanthin, cryptocapsin, capsanthin, capsoneoxanthin	Aril: 226, Mesocarp: 83.4 fw	[75]
Ericaceae	*Arbutus unedo*	(all-E)-Violaxanthin, 9Z-violaxanthin, (all-E)-neoxanthin, (9′Z)-neoxanthin, lutein	>340 dw	[83]
Elaeagnaceae	*Elaeagnus umbellata*	β-carotene, lutein, lycopene	1851 dw	[84]
	*Hippophae rhamnoides*	α- and β-carotene, lutein, cryptoxanthin, zeaxanthin, lycopene	531–967 dw	[85]
Myrtaceae	*Eugenia stipitata*	Lutein, β-cryptoxanthin, zeinoxanthin, β-carotene	Peel: 24.84; Pulp: 8.06	[86]
	*Eugenia uniflora*	β-carotene	5.86 fw	[87]
	*Psidium cattleianum*	β-carotene	6.27 fw	[87]
Rosaceae	*Cerasus humilis*	β-carotene, zeaxanthin, lutein, violaxanthin	9.5–28.2 fw	[88]
	*Crataegus monogyna*	Mutatoxanthin, lutein, α-cryptoxanthin, β-cryptoxanthin, cis-β-carotene, all-trans-β-carotene, lycopene	420 dw	[77]
	*Rosa canina*	Lycopene, β-carotene, β-cryptoxanthin, lutein	224 fw	[76]
	*Various* (e.g., *Rubus*, *Sorbus*)	α-, β-, γ-carotene, β-cryptoxanthin, zeaxanthin, violaxanthin, lutein, lycopene	Highly variable	[89]
Sapotaceae	*Pouteria torta*	Lutein, lutein epoxide, β-carotene, β-carotene epoxide, violaxanthin	11.28 fw	[85,90]
Solanaceae	*Lycium ruthenicum*	β-carotene, β-cryptoxanthin, zeaxanthin, neoxanthin, violaxanthin, lutein	2.4 dw	[91]

### 4.3. Tocols (Vitamin E)

**Figure 3:**
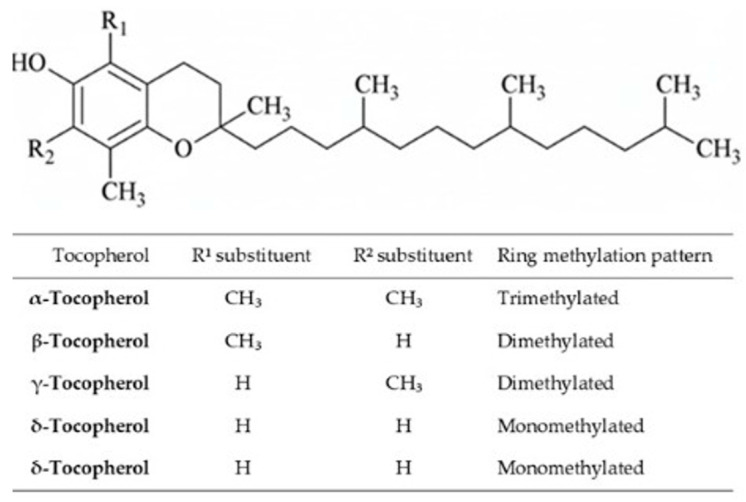
Tocopherol chemical structures. The table indicates the number and position of methyl groups on the aromatic ring.

Tocols, collectively referred to as vitamin E, comprise four tocopherols (α-, β-, γ-, and δ-) and four corresponding tocotrienols (Figure 3). These lipid-soluble compounds play a central role in protecting cellular membranes and lipoproteins from oxidative damage by interrupting lipid peroxidation chain reactions. Beyond their antioxidant function, tocols modulate immune responses, inflammation, and cell signalling pathways, contributing to the prevention of chronic diseases [92,93].

Among the different isoforms, α-tocopherol is the most biologically active and preferentially retained in human tissues due to selective hepatic transport mechanisms. Adequate vitamin E intake has been associated with reduced oxidative stress and a lower risk of cardiovascular and neurodegenerative disorders [93,94]. While plant oils and nuts are major dietary sources, several WEFs can meaningfully contribute to vitamin E intake, particularly in traditional diets.

The data presented in Table 4 reveal substantial interspecific variability in total tocol content among WEFs. Total tocol concentrations range from trace amounts in *C. albidum* (0.02 mg/100 g fw) to comparatively high levels in *R. micrantha* (19.64 mg/100 g dw), *R. ulmifolius* (13.48 mg/100 g fw), and *H. rhamnoides* subsp. *sinensis* (10.2 mg/100 g fw). α-tocopherol is the dominant isoform, accounting for the majority of total tocols, as observed in *G. atroviridis*, *C. monogyna*, and *R. canina*.

Several WEFs also contain appreciable levels of γ- and δ-tocopherols, particularly *R. ulmifolius* and *P. spinosa*, which may enhance antioxidant efficacy through complementary mechanisms. Small amounts of tocotrienols have been reported in some species (Table 4), although data on these compounds remain scarce. This highlights an important knowledge gap, given the emerging evidence for tocotrienol-specific benefits, including cholesterol-lowering and neuroprotective effects [92,93].

Antioxidant activity data reported alongside tocol content further support the functional relevance of vitamin E in WEFs. Species such as *C. monogyna*, *R. ulmifolius*, and *P. spinosa* exhibit relatively low EC_50_ values in β-carotene bleaching and TBARS assays, indicating effective inhibition of lipid peroxidation and reinforcing the contribution of tocols to overall antioxidant capacity [95,96].

The nutritional impact of tocols from WEFs depends on bioavailability, which is influenced by the lipid content of the fruit matrix and concurrent dietary fat intake. Processing and storage conditions also affect tocol stability, as exposure to heat, oxygen, and light can reduce vitamin E content [92,93]. These factors should be considered when promoting WEFs as dietary vitamin E sources or developing value-added products.

**Table 4:** Tocol content (mean values) of the pulp of selected Wild Edible Fruits.

Family	Species	Tocols (mg/100 g fw)					Reference
		Total Tocols	α-Tocopherol	β-Tocopherol	γ-Tocopherol	δ-Tocopherol	
Clusiaceae	*Garcinia atroviridis*	7.56	7.56				[97]
Combretaceae	*Terminalia ferdinandiana*	1.04	1.02		0.02		[98]
Cunoniaceae	*Davidsonia pruriens*	0.09	0.04		0.03	0.02	[98]
Elaeagnaceae	*Hippophae rhamnoides*	4.01	3.05	0.26	0.73		[99]
	*H. rhamnoides* subsp. *sinensis*	10.2	8.5	1.0	6.0	1.0	[100]
Ericaceae	*Vaccinium vitis-idaeaa*	2.14	1.53		0.13		[99]
Myrtaceae	*Syzygium luehmannii*	0.46	0.23		0.23		[98]
	*Psidium guajava*	0.88					[97]
Rosaceae	*Crataegus monogyna*	3.37	2.88	0.15	0.17	0.16	[95]
	*Prunus spinosa*	3.58–5.41	2.81–5.23	0.02–0.08	0.09–0.75	0.00–0.04	[95,96]
	*Ribes uva-crispa*	0.84	0.73		0.11		[99]
	*Rosa canina*	4.38–4.29	3.63–4.14	0.10–0.14	0.10–0.56		[96,99]
	*R. micrantha*	19.64 dw	18.17 dw		1.47 dw		[101]
	*Rubus chamaemorus*	3.62	2.95	0.20	0.45	0.02	[99]
	*Rubus ulmifolius*	13.48	3.38	0.24	3.73	3.69	[95]
Rutaceae	*Acronychia acidula*	0.58	0.28		0.01	0.29	[98]
	*Citrus australasica*	2.38	2.34		0.04		[98]
	*Citrus glauca*	0.78	0.70		0.08		[98]
Santaleaceae	*Santalum acuminatum*	1.30	1.17		0.09	0.04	[98]
Sapotaceae	*Chrysophyllum albidum*	0.02 ^a^					[102]
Solanaceae	*Lycium ruthenicum*	0.65–0.10 dw	0.52–0.56 dw		0.13–0.42 dw	0.00–0.04 dw	[91]

^a^ Small amounts of α- and γ-tocotrienols.

### 4.4. Sterols and Triterpenoids

Sterols and triterpenoids are structurally related lipophilic phytochemicals widely distributed in WEFs. Phytosterols—primarily β-sitosterol, campesterol, and stigmasterol—are plant analogues of cholesterol that modulate lipid metabolism, while triterpenoids such as ursolic, oleanolic, and betulinic acids exhibit diverse biological activities, including anti-inflammatory, antioxidant, and anticancer effects [103,104].

From a nutritional standpoint, phytosterols reduce intestinal cholesterol absorption by competing with dietary cholesterol for incorporation into mixed micelles, thereby lowering plasma LDL cholesterol and reducing cardiovascular risk [105]. Although vegetable oils and seeds are major sterol sources, WEFs can provide meaningful contributions in traditional diets where access to fortified foods is limited.

**Figure 4:**
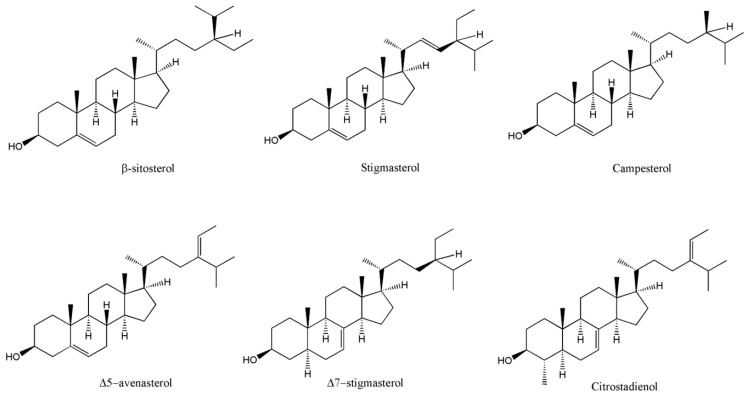
Sterols commonly present in Wild Edible Fruits.

The sterol profiles summarised in Table 5 demonstrate substantial variability among WEF species. Total sterol contents range from relatively low levels in *A. martinii* (13.92 mg/kg dw) and *H. thebaica* (29.9 mg/kg dw) to notably high concentrations exceeding 900 mg/kg dw in species such as *V. lentago*, *R. cathartica*, *R. hirtellum*, and *V. trilobum* [106]. Across most taxa, β-sitosterol is the dominant compound, consistent with its established role as the principal cholesterol-lowering phytosterol. Figure 4 shows the sterols commonly found in WEFs.

Several Rosaceae species (*A. alnifolia*, *A. melanocarpa*, *R. idaeus*, *Ribes* spp.) exhibit consistently high sterol levels, indicating that this family represents a particularly rich source of sterols among WEFs. Wild olives (*O. europaea*) also contain measurable sterol concentrations, reinforcing their cardiometabolic relevance even in non-cultivated forms [107]. In contrast, species reported without quantitative data highlight gaps in sterol profiling rather than an absence of these compounds.

Triterpenoids complement the nutritional effects of sterols by contributing to pharmacological activities beyond lipid-lowering. Ursolic and oleanolic acids, frequently concentrated in fruit peels and waxy cuticles, have been shown to modulate inflammatory pathways, oxidative stress, glucose metabolism, and apoptosis [103,108]. Although triterpenoids are often quantified together with sterols (Table 5), their specific contribution remains insufficiently characterised in many WEFs.

The bioavailability of sterols and triterpenoids is limited by their hydrophobic nature; however, repeated dietary intake, food processing that enhances extractability, and synergistic interactions with phenolics and tocols can increase their physiological impact [109,110]. Traditional processing practices, such as drying or fermenting, may further influence their stability and bioactivity.

**Table 5:** Sterol profiles of the pulp of selected Wild Edible Fruits.

Family	Species	Sterols	Total Sterols (mg/kg)	Reference
Adoxaceae	*Sambucus nigra*	β-sitosterol, campesterol, stigmasterol, isofucosterol	543.89 dw	[81,107,111]
	*Viburnum lentago*	Stigmasterol, campesterol, β-sitosterol, isofucosterol	1024 dw	[106]
	*Viburnum trilobum*	Stigmasterol, campesterol, β-sitosterol, isofucosterol	905 ^a^ dw	[106]
Arecaceae	*Hyphaene thebaica*	β-sitosterol, campesterol, stigmasterol, ergosterol, avenasterol	29.9 dw	[112]
Apocynaceae	*Carissa carandas*	β-sitosterol, campesterol, stigmasterol	139.2 fw	[81,107]
Caprifoliaceae	*Lonicera caerulea*	β-sitosterol, campesterol, cholesterol, cycloartanol, stigmasterol, isofucosterol	46.61 fw/969 ^a^ dw	[106,113]
	*Symphoricarpos albus*	β-sitosterol, stigmasterol	683 ^a^ dw	[106]
Ebenaceae	*Diospyros mespiliformis*	β-sitosterol, campesterol, stigmasterol, ergosterol, avenasterol	29.2 dw	[112]
Ericaceae	*Vaccinium angustifolium*	Stigmasterol, campesterol, β-sitosterol, isofucosterol	670 ^a^ dw	[106]
Fabaceae	*Detarium senegalense*	β-sitosterol, campesterol, stigmasterol, ergosterol, avenasterol	32.1 dw	[112]
Oleaceae	*Olea europaea* (wild olives)	β-sitosterol, campesterol, stigmasterol	1.114 dw	[107]
Rhamnaceae	*Rhamnus cathartica*	β-sitosterol, campesterol	1016 ^a^ dw	[106]
Rosaceae	*Amelanchier alnifolia*	Stigmasterol, β-sitosterol, isofucosterol	865 ^a^ dw	[106]
	*Aronia melanocarpa*	Stigmasterol, campesterol, β-sitosterol, isofucosterol	612 ^a^ dw	[106]
	*Prunus virginiana*	Stigmasterol, campesterol, β-sitosterol, isofucosterol	283 ^a^ dw	[106]
	*Ribes hirtellum*	Stigmasterol, campesterol, β-sitosterol, isofucosterol	938 ^a^ dw	[106]
	*Ribes rubrum*	Stigmasterol, campesterol, β-sitosterol, isofucosterol	646 ^a^ dw	[106]
	*Ribes rubrum*	Stigmasterol, campesterol, β-sitosterol, isofucosterol	718 ^a^ dw	[106]
	*Rubus idaeus*	Stigmasterol, campesterol, β-sitosterol, isofucosterol	614 ^a^ dw	[106]
Solanaceae	*Lycium barbarum* (Goji berries)	β-sitosterol, campesterol, Δ5-avenasterol	-	[114]
Umbelliferae	*Buplerum croceum*	β-sitosterol, stigmasterol, campesterol, Δ5-avenasterol, Δ-5,24-stigmastadienol	-	[115]
	*Buplerum flavum*	β-sitosterol, stigmasterol, campesterol, Δ5-avenasterol, Δ-5,24-stigmastadienol	-	[115]
	*Buplerum rotundifolium*	β-sitosterol, stigmasterol, campesterol, Δ5-avenasterol, Δ-5,24-stigmastadienol	-	[115]
Vitaceae	*Ampelocissus martinii*	β-sitosterol, stigmasterol	13.92 dw	[116]
	*Vitis riparia*	Stigmasterol, campesterol, β-sitosterol	469 ^a^ dw	[106]

^a^ Sterols + terpenes.

### 4.5. Vitamin C

There are two acid vitamers with vitamin C activity, ascorbic and dehydroascorbic acids; the former is highly active. Vitamin C is an essential water-soluble micronutrient with well-established roles in antioxidant defence, collagen synthesis, immune function, neurotransmitter production, and iron absorption. Because humans cannot synthesise vitamin C endogenously, fruits represent the primary dietary source [117,118].

WEFs are frequently richer sources of vitamin C than many cultivated fruits. As summarised in Table 6, reported ascorbic acid contents range widely, from modest levels below 20 mg/100 g in *P. spinosa* and *C. monogyna* to high concentrations exceeding 400 mg/100 g in species such as *P. emblica*, *R. pratincola*, *A. unedo*, and *A. marmelos*. In these fruits, small serving sizes can meet or exceed recommended daily intakes, highlighting their nutritional efficiency [42,59,119,120].

Several taxa consistently emerge as vitamin C–dense WEFs. *P. emblica* shows particularly broad variability (33–437 mg/100 g), reflecting differences in genotype, maturity, and analytical approach, yet confirming its status as a globally recognised vitamin C–rich fruit. *A. digitata* may contain up to 210.4 mg/100 g of vitamin C [121]. Similarly, other *Rosa* species, *V. opulus*, and *A. occidentale* display high ascorbic acid levels, reinforcing their importance in traditional diets across Africa, Asia, and Europe [65,122,123].

Biologically, vitamin C acts as a direct scavenger of reactive oxygen species and regenerates oxidised vitamin E, strengthening antioxidant networks within the fruit matrix and in human tissues. Adequate intake has been associated with reduced cardiovascular risk, improved immune responsiveness, enhanced wound healing, and potential protection against infections and inflammatory disorders [117,118]. These effects are particularly relevant for WEFs that combine high vitamin C with phenolics and carotenoids, amplifying overall antioxidant capacity.

The data in Table 6 also reveal strong geographic and ecological patterns. Many African and Asian WEFs (*A. marmelos*, *A. digitata*, *D. caffra*, *H. rhamnoides*) combine high vitamin C with mineral and phytochemical richness, supporting their role in addressing micronutrient deficiencies in resource-limited settings. Moreover, vitamin C enhances non-heme iron absorption, an interaction of particular importance in predominantly plant-based diets [117].

Vitamin C content is highly sensitive to environmental factors, ripening stage, and post-harvest handling. Heat, oxygen exposure, and prolonged storage can substantially decrease ascorbic acid levels, making fresh or minimally processed consumption of WEFs nutritionally advantageous [110,118].

**Table 6:** Ascorbic acid content of the pulp of selected Wild Edible Fruits.

Family	Especies	Range	Ascorbic Acid (mg/100 g)	Additional Notes	Reference
Adoxaceae	*Viburnum opulus*	Eurasia NW Africa	185.4	Rich in antioxidants like phenolic, giving high antioxidant, anti-inflammatory, and cardioprotective properties	[123]
Anarcadiaceae	*Anacardium occidentale*	Northeastern Brazil and Southeastern Venezuela	202	High antioxidant activity	[120]
	*Sclerocarya cafra*	Transvaal, South Africa	67.9	Rich in minerals	[122]
	*Spondias dulcis*	Tropical Asia	51.2	Rich in carotenes, fiber, and antioxidants. Improves digestion, immunity, and skin health	[120]
Apocynaceae	*Carissa macrocarpa*	South Africa	74.1	Rich in anthocyanins, calcium, magnesium, and phosphorus	[122]
Caprifoliaceae	*Lonicera caerulea* subsp. *altaica*	Circumpolar regions	42.7	Rich in antioxidants like anthocyanins, polyphenols, and flavonoids, offering benefits for heart health, vision, inflammation, blood sugar, and potentially cancer prevention	[123]
	*Landolphia capensis*	Southern Africa	60.1	Antioxidant, antimicrobial, and potential medicinal (anti-inflammatory, antimalarial) properties	[122]
Elaegnaceae	*Hippophae rhamnoides*	Eurasia	66.64	Rich in vitamins, minerals, antioxidants, essential fatty acids, and polyphenols. Offering strong antioxidant, anti-inflammatory, cardioprotective, and antimicrobial properties	[123]
Ericaceae	*Arbutus unedo*	Mediterranean area	182–419	High vitamin C and phenolic content; strong antioxidant activity	[42,59]
Malvaceae	*Adansonia digitata*	Central and Southern Africa	213	High in phenolics, acting as an antioxidant, anti-inflammatory, and prebiotic	[122]
Moraceae	*Morus alba*	Himalaya	29.53	High in phenols and flavonoids, reported to be beneficial against lipid and lipoproteins and to delay the onset of atherosclerosis	[42]
Oxalidaceae	*Averrhoa carambola*	South-East Asia	25.5	Low in calories and high in vitamin C, fibre, and antioxidants. Supports immune health, aids digestion, and helps with hydration	[120]
Phyllanthaceae	*Phyllanthus emblica*	South-East Asia to southern China	33.1–437	Rich in vitamin C; widely recognised for strong antioxidant capacity and traditional medicinal use	[19,42,120]
Rosaceae	*Crataegus monogyna*	Europe and western Asia	15.2	Rich in antioxidants, vitamins, minerals, and fibre. It offers cardioprotective, antioxidant, anti-inflammatory, and hypotensive properties	[95]
	*Prunus spinosa*	Europe	7.73	Rich in antioxidants, minerals, fibre, and tannins, giving it astringent, anti-inflammatory, diuretic, and antimicrobial properties	[95]
	*Rosa pratincola*	North America	426	Rich in antioxidants (polyphenols, flavonoids, carotenoids), and vitamins A, E, K, providing anti-inflammatory, immune-boosting, antioxidant, and skin-health benefits	[119]
	*Rubus idaeus*	Ubiquitous	26.4	Rich in antioxidants and nutrients, offering anti-inflammatory, antimicrobial, astringent, and diuretic properties	[119]
	*Rubus ulmifolius*	Ubiquitous	6.0–26.8	Lower vitamin C than *A. unedo*, but rich in phenolic acids and anthocyanins	[59,95]
Rutaceae	*Aegle marmelos*	Indian subcontinent and Southeast Asia	517	Rich in phytochemicals: tannins, flavonoids, and phenolic compounds. Antioxidant, anti-inflammatory, antimicrobial, and antidiabetic	[120]
Salicaceae	*Dovyalis caffra*	Southern Africa	117	Rich in antioxidants, fiber, potassium, and calcium	[122]
Sapotaceae	*Chrysophyllum albidum*	Central and Western Africa	86.8–99.6	Rich in antioxidants, minerals (calcium, iron), fiber, flavonoids and phenols. Anti-inflammatory, anti-diabetic, and potentially aid cognitive function	[102]

### 4.6. Essential Minerals

WEFs represent rich and diverse sources of essential dietary minerals, emphasising potassium, calcium, magnesium, iron, zinc, copper, and manganese, all of which are fundamental to metabolic regulation, skeletal integrity, oxygen transport, immune competence, and oxidative stress control. Unlike macronutrients, these micronutrients primarily function as enzyme cofactors and signalling mediators, making their adequate intake critical for sustaining long-term metabolic health and physiological homeostasis [124,125].

Macrominerals such as potassium and sodium play key roles in maintaining electrolyte balance, nerve impulse transmission, and muscle contraction, whereas calcium and magnesium are essential for bone mineralisation, vascular function, and several enzymatic reactions. Trace elements—including iron, zinc, copper, and manganese—support oxygen transport, DNA synthesis, immune regulation, and antioxidant defence through their involvement in metalloenzymes and redox systems [126,127,128]. In addition to macronutrients, WEFs are important sources of micronutrients that are often deficient in local diets.

The mineral composition data summarised in Table 7 demonstrate pronounced interspecific variability, which permits combining wild fruits with complementary mineral profiles to meet diverse nutritional requirements. In addition, numerous WEFs could also be highlighted as mineral-dense foods. Among these, *C. welwitschii* (Achariaceae) stands out due to its extraordinarily high magnesium content (~1.25 g/100 g dw) and manganese levels (1.75 mg/100 g), together with substantial potassium and zinc contents. Such a profile suggests strong potential to support electrolyte balance, enzymatic function, and antioxidant defences even at modest consumption levels [129].

Several species from the Anacardiaceae and Annonaceae families—including *P. microcarpa*, *S. birrea*, and *A. senegalensis*—exhibit well-balanced mineral profiles, providing meaningful amounts of iron, zinc, manganese, potassium, and calcium. With iron concentrations frequently exceeding 1 mg/100 g and zinc reaching up to 640 µg/100 g, these fruits are particularly relevant for supporting haematological health and immune function in plant-based and resource-limited dietary contexts [129,130].

Markedly high iron levels are observed in several taxa, notably *B. aethiopum* (Arecaceae), where iron content reaches up to 5.66 mg/100 g, accompanied by appreciable copper concentrations that facilitate iron metabolism and redox homeostasis. Similarly, species such as *B. aristata* and related *Berberis* taxa show high iron and calcium levels, reinforcing their traditional nutritional and medicinal significance [131]. In parallel, *O. polyacantha* (Cactaceae) combines elevated calcium (180 mg/100 g) with high manganese content, suggesting benefits for bone health and antioxidant enzyme activity [119].

Other WEFs contribute targeted mineral strengths that further enhance dietary diversity. *C. jambhiri* (Rutaceae) is notable for its high zinc (>2.5 mg/100 g) and calcium content, supporting immune competence and skeletal health. Species from the Moraceae, Sapotaceae, and Malvaceae families—such as *M. nigra*, *C. albidum*, and *A. digitata*—exhibit broad-spectrum mineral profiles, including potassium, calcium, magnesium, and trace elements essential for metabolic resilience [6,102].

**Table 7:** Mineral composition (dry weight) of the pulp of selected Wild Edible Fruits.

*Family*	*Species*	µg/100 g			mg/100					Reference
		Cu	Mn	Zn	Fe	K	Na	Ca	Mg	
Achariaceae	*Caloncoba welwitschii*	310	1750	560	0.8	451.3	14.4	76.1	1253.5	[129]
Anacardiaceae	*Pseudospondias microcarpa*	70	600	280	1.28	425.6	1.05	18.1	24.8	[129]
	*Sclerocarya birrea*	100	110	340–700	1.12–3	2753	15.2–30	36.2–481	138–310	[130,132,133]
	*Spondias pinnata*	3	–	12	0.22	13	163	189	45	[63]
Annonaceae	*Annona senegalensis*	170	430	640	1.33	-	-	28.9	42.2	[130]
	*Annona stenophylla*	150	420	210	0.6	435.4	3.85	59	28.8	[129]
Apocinaceae	*Landolphia buchananii*	190	950	260	0.47	281.8	3.61	10.4	16.6	[129]
	*Landolphia camptoloba*	190	2670	390	0.72	274.8	-	15.9	25.5	[129]
	*Landolphia congolensis*	280	330	460	1.24	340.4	9.3	5.35	10.8	[129]
	*Landolphia dewevrei*	170	410	230	1.36	448.9	-	6.56	11.8	[129]
	*Landolphia lanceolata*	230	270	160	0.5	297.4	1.48	4.21	11.3	[129]
	*Landolphia owariensis*	170	270	350	0.49	164.7	4.45	5.71	14	[129]
	*Landolphia robustior*	250	1210	300	0.77	210.4	3.44	10.7	16	[129]
Arecaceae	*Borassus aethiopum*	720	290	190	2.05–5.66			44–108.3	20.6–31.7	[134,135]
	*Butia capitata*	100	100	100	0.7	293.9	0.9	3	6.3	[136]
	*Parkia biglobosa*	488–970	3340–5440	1150–1260	0.74–3.1	1997	77.8	145.3–284	4.5–202	[134,137,138]
	*Rafia matombe*	80	4950	570	0.43	242.9	5.72	251.6	56	[129]
Berberidaceae	*Berberis aristata*	2300	-	3.7	52.3	392.8	47.1	396.3	5.1	[131]
	*Berberis asiática*	2000	-	11.2	180.8	474.6	72.6	872.5	5.8	[131]
	*Berberis jaeschkeana*	2500	-	4.3	132	648.9	66.3	359.9	5.6	[131]
	*Berberis lycium*	1600	-	2.54	36.5	432.6	31.7	190.2	1.5	[131]
	*Berberis pseudumbellata*	1500	-	1.6	12.3	432.6	24	158.4	0.56	[131]
Cactaceae	*Hylocereus triangularis*	15	11	34	0.5	207	8	31	23	[139]
	*Opuntia polyacantha*	100	1560	611	1.15	130	<9	180	69	[119]
Caricaceae	*Carioca papaya*	1	3	9	37	85	7	16	10	[139]
	*Vasconcella pulcra*	22–77	–	40–51.5	7.04–8.06	598.5–658.9	22.6–32.6	17.6–20.0	34.4–35.4	[140]
	*Vasconcella x heibornii*	<0.5	–	83–156	6.31–6.61	371.1–417.9	6.31–6.61	13.5–18.2	31.0–35.6	[140]
Chrysobalanaceae	*Chrysobalanus icaco*	125	240	55	0.37	186.5	62.5	5.25	27.5	[141]
	*Parinari capensis*	<130	210	110	0.26	222.3	1.81	80.7	26.7	[129]
Clusiaceae	*Garcinia mangostana*	83.55	176.1	116.6	0.34	23.07	–	10.43	18.68	[142]
	*Garcinia xanthocymus*	3490	2080	2920	10.89	30	4.94	14.03	31.62	[6]
Combretaceae	*Terminalia chebula*	275.5	247.2	1029	0.76	358.4	4.57	10.87	–	[4]
Flacourtiaceae	*Flacourtia jangomas*	29.79	268	865.6	0.07	17.61	–	5.1	10.37	[142]
Lamiaceae	*Vitex madiensis* subsp. *Madiensis*	100	4700	310	360	504	3.07	26.4	25.6	[129]
Loganiaceae	*Strychnos cocculoides*	30	1170	40	0.16	206.2	-	17.1	28.1	[129]
	*Strychnos pungens*	160	1340	110	0.26	563	-	21.9	36.8	[129]
Malpighiaceae	*Malpighia glabra*	4	9	19	0.47	202	<0.1	38	56	[139]
Malvaceae	*Adansonia digitata*	550–6000	390–6000	0.01–2400	0.017–4.40	2308–2392	0.054–5.5	3.4–387	2.1–209	[130,132,133,134,143]
	*Cola parchycarpa*	-	-	1250	2.66	83.7	45.3	163.8	48.5	[144]
	*Cola Rostrata*	-	-	1410	2.51	82.7	42.7	170.3	80.4	[144]
Melastomataceae	*Clidemia rubra*	10	9.61	63	1.73	163.4	0.85	43.6	9.21	[145]
	*Tristemma mauritianum*	190	2650	440	1.42	162.	1.85	137	37.2	[129]
Moraceae	*Artocarpus heterophyllus*	1450	11.4	5410	1.13	410	44.3	27.1	28.3	[6]
	*Morus nigra*	450	4270	6420	4	2190	69.2	474.4	163	[6]
	*Artocarpus lacucha*	–	755.3	336.8	0.28	137.55	–	26.17	8.8	[142]
	*Streblus taxoides*	73.87	297	30.16	0.45	39.45	2.79	4.75	–	[4]
Myrtaceae	*Acca sellowiana*	160	62	22	0.4	68.4	0.4	6.8	3.9	[136]
	*Eugenia involucrata*	37	100	100	0.4	124.9	4.1	9.8	6.7	[136]
	*Eugenia myrcianthes*	19	100	100	0.2	112.4	0.3	5.1	7.2	[136]
	*Eugenia malaccensis*	3	6	7	0.15	164	10	15	25	[139]
	*Eugenia rothii*	161.7	3128	457	1.44	676.6	8.64	20.5	–	[4]
	*Eugenia stipitata*	7	8	18	0.38	78	2	25	38	[139]
	*Eugenia uniflora*	7	11	19	0.49	165	<0.1	48	38	[139]
	*Gaylussacia brasiliensis*	100	–	400	6.03	115.4	13.21	58.23	21.9	[146]
	*Myrciaria cauliflora*	6	28	19	0.33	213	5	22	16	[139]
	*Psidium guajava*	8	9	20	0.28	332–366	5–7	20–29	12–17	[139]
Passifloracea	*Passiflora edulis*	5–6	12–16	20–43	0.61–0.66	100–764	16–30	22–53	16–26	[139]
	*Passiflora foetida*	260	530	870	0.84	484	1.81	86.6	55.2	[129]
Phyllanthaceae	*Antidesma velutinosum*	110	–	430	0.58	11	230	325	115	[63]
	*Antidesma venosum*	180	2380	360	0.9	378	3.65	142	46.6	[129]
	*Baccaurea ramiflora*	23.6	103.7	85.8	0.14	18.8	–	4.95	10.7	[142]
	*Phyllanthus acidus*	200–489.9	1092–1913	0.8	1.48–1.86	104.4–302.4	8.96–10.8	11.3–22.4	–	[4,142]
	*Phyllanthus emblica*	4	–	140	0.16	13	151	42	13	[63]
Polygonaceae	*Oxygonum fruticosum*	40	510	280	0.97	140.6	-	17.1	20.8	[129]
Rhamnaceae	*Ziziphus jujubar*	10	13	66	3.37	107	4	385	11	[139]
	*Ziziphus* *mauritiana*	70.0	214.2	201.8	0.42	41.2	2.07	6.48	–	[4,147,148]
	*Ziziphus* *oenophia*	554.8	–	110.0	1.82	308.7	11.21	40.6	–	[4]
Rosaceae	*Prunus americana*	35	76	94	0.174	364	<9	11	8	[119]
	*Prunus jenkinsiii*	2950	21,510	5350	3.07	2850	42.4	74.6	226.7	[6]
	*Prunus virginiana*	186	417	328	0.685	379	<9	60	27	[119]
	*Rosa pratincola*	113	1.02	245	1.06	429	<9	169	69	[119]
	*Rubus idaeus*	100	1.56	611	1.15	130	<9	36	691	[119]
	*Rubus treutleri*	3.7	20,680	4480	11.2	1190	136.05	169.7	241.8	[6]
Rubiaceae	*Morinda citrifolia*	11	28	21	0.57	374	13	43	17	[139]
	*Morinda tinctoria*	145.1	1.16	0.31	0.5	78.8	3.7	10.6	–	[4]
	*Sabicea gilletii*	230	5670	240	1.03	143.1	2.1	63.9	30.2	[129]
Rutaceae	*Aegle marmelos*	751.3	75.31	160.3	0.82	158.7	–	26.1	6.19	[142]
	*Citrus jambhiri*	1010	610	2570	2.11	220	51.5	323.7	136.8	[6]
	*Glycosmis pentaphylla*	725.6	1547	1129.8	2.02	258.8	11.9	34.0	–	[4]
	*Toddalia asiatica*	12.0	213.2	49.27	12.26	218.63	10.02	25.0	–	[4]
Sapindaceae	*Litchi chinensis*	25.53	238.9	297.31	0.05	31.19	0.18	0.91	–	[4]
Sapotaceae	*Chrysophyllum albidum*	5630–5820	4850–5160	8240–8270	2.23–2.29	666.2–700.8	35.5–54.5	365.5–425.0	–	[102]
	*Mimusops elengi*	228.8	3.58	0.94	2.11	362.5	23.8	886.0	–	[4]
Solanaceae	*Cyphomandra betacea*	11	20	2	0.41	524	6	26	20	[139]
	*Solanum sisymbrifolium*	67	67	100	0.6	256.9	5	14.7	14.4	[145]
	*Solanum torvum*	560	3072	1395.0	2.13	304.7	13.0	59.9	–	[4]
Zingiberaceae	*Aframomum alboviolaceum*	16.9	210	0.75	0.76	453.9	1.24	16.9	32.4	[129]
	*Aframomum angustifolium*	27.5	350	1.25	0.97	464.7	1.01	27.5	59.5	[129]
	*Aframomum giganteum*	19.4	340	1.25	1.44	455.3	1.37	19.4	53.9	[129]
Zygophyllaceae	*Balanites aegyptiaca*	620	650	2920	5.8	-	-	120	81.4	[134]

### 4.7. Terpenoids and Essential Oils

**Figure 5:**
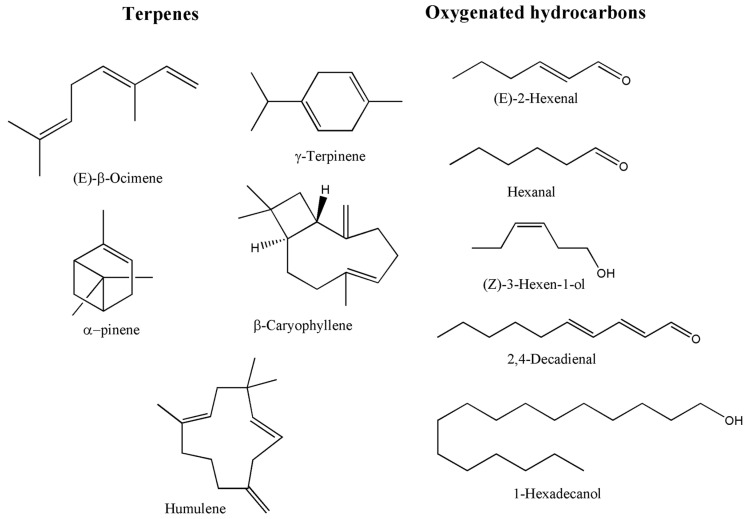
Chemical structure of some terpenoid components of EOs present in Wild Edible Fruits.

WEFs contain a wide variety of terpenoids and essential oils (EO) that contribute to their nutritional, medicinal, and sensory properties. Terpenoids—also referred to as terpenes—represent the largest and most structurally diverse class of plant secondary metabolites. They are biosynthesised from isoprene units and are classified into mono-, sesqui-, di-, tri-, and tetraterpenes according to carbon number and structural complexity [149] (Figure 5). These compounds are widely distributed in aromatic plants and fruits, including citrus species, tea, thyme, and sage, as well as numerous wild fruit taxa.

The principal terpenoid constituents identified in selected WEF species are summarised in Table 8. Several WEFs are particularly rich in terpenoids. Fruits of *Z. armatum*, for example, contain high levels of monoterpenes, with linalool identified as a dominant constituent [150]. Similarly, species from the Annonaceae, Clusiaceae, and Moraceae families produce complex mixtures of mono- and sesquiterpenes alongside triterpenoids such as lupeol, ursolic acid, oleanolic acid, and betulinic acid, which are compounds widely recognised for their bioactive potential [151,152,153].

Essential oils are volatile, lipophilic mixtures predominantly composed of terpenes and terpenoids, typically extracted from fruits, leaves, or seeds. The essential oil of *S. terebinthifolius* fruit is characterised by a high proportion of monoterpenes—particularly δ-3-carene, limonene, α-phellandrene, and α-pinene—together with smaller amounts of sesquiterpenes such as trans-caryophyllene [154]. Essential oil yield can vary substantially among species and populations; in *Z. armatum*, yields ranging from 2.72% to 7.6% have been reported, with higher concentrations observed in wild populations from elevated altitudes [150].

From a biological perspective, terpenoids and EOs derived from WEFs exhibit a broad spectrum of bioactivities. Antibacterial effects have been demonstrated for *S. terebinthifolius* fruit essential oil against hospital-derived bacterial strains [154], while antioxidant, antimicrobial, and anti-inflammatory properties are consistently reported across multiple taxa [155]. At the molecular level, terpenoids are known to exert antiplasmodial, antiviral, anticancer, and antidiabetic activities, largely through modulation of oxidative stress, inflammation, and metabolic signalling pathways [149,156].

Traditionally, WEFs rich in terpenoids and EOs have been used to treat gastrointestinal disorders, respiratory ailments, inflammatory conditions, and cardiovascular complaints [19,20]. Beyond their medicinal relevance, these compounds also play a crucial role in defining the aroma and flavour profiles of wild fruits, making them valuable to the food, flavour, and fragrance industries. Terpenoids are widely exploited in the formulation of natural flavourings, fragrances, nutraceuticals, and pharmaceutical products due to their volatility, bioactivity, and consumer appeal [156,157,158,159].

**Table 8:** Terpenoids and EOs of the pulp of selected Wild Edible Fruits.

Family	Fruit	Main Terpenoids	Main Essential Oils/ Key Components	Reference
Anacardiaceae	*Schinus terebinthifolia*	δ-3-carene, limonene, α-phellandrene, α-pinene, trans-caryophyllene	Monoterpenes (85.8%), Sesquiterpenes (5.3%) (% of total EO)	[154]
Annonaceae	*Annona* species	α-Pinene, β-Pinene, Limonene, 1,8-Cineole, Linalool, α-Terpineol, Geraniol, Nerol, Citronellol, β-Caryophyllene, α-Humulene, Germacrene D, δ-Cadinene, Spathulenol, Ledol, α-Cadinol, β-Selinene, Eudesmol, Lupeol, Ursolic acid, Oleanolic acid, Betulinic acid, Uvaol, Maslinic acid, Pomolic acid	Present in its EOs	[153]
Apiaceae	*Levisticum officinale*	Z-ligustilide, β-phellandrene, α-terpinyl acetate	Z-ligustilide (35.1%), β-phellandrene (34.4%), α-terpinyl acetate (4.2%)	[160]
Bromeliaceae	*Greigia sphacelata*	Euonyminol, Monic acid A, Dictamnoside N, Marrubiin, Quillaic acid	Present in its EOs	[161]
Burseraceae	*Dacryodes edulis*	Sabinene, terpinene-4-ol, α-pinene, p-cymene	Present in its EOs	[162]
Clusiaceae	*Garcinia morella*	Ursolic acid, Betulinic acid, Alloaromadendrene, Aromadendrene, Ascaridole, Caryophyllene oxide, Germacrene B, Globulol, Myrcene, Selina-3,7(11)-diene, Spathulenol, α-Copaene, α-Humulene, β-Caryophyllene, β-Copaene, β-Gurjunene, δ-Amorphene, δ-Elemene	Rich in terpenoids, including xanthonoids and triterpenoids	[152]
Fabaceae	*Copaifera langsdorffii*	Germacrene D, bicyclogermacrene, trans-caryophyllene, δ-elemene	Non-oxygenated sesquiterpenes	[163]
Moraceae	*Brosimum gaudichaudii*	Convallatoxin, Maragenin I acetate, Moruslanosteryl acetate	Present in hydroethanolic extracts	[164]
	*Ficus hispida*	Lupeol acetate, β-Amyrin acetate, β-Amyrin, β-Sitosterol, Gluanol, Oleanolic acid, α-Amyrin, Ficustriol, Linalool, Linalool oxide, Terpineol, 2,6-Dimethyl-1,7-octadiene-3,6-diol	Included among various bioactive compounds	[151,152]
Rutaceae	*Zanthoxylum armatum*	Linalool, cinnamate (E)methyl, limonene, myrcene, sabinene and terpinen-4-ol	High percentage of linalool (74.12%)	[150]

### 4.8. Polysaccharides and Dietary Fibres

WEFs are highly valued for their high content of dietary fibres and polysaccharides, which improve the functioning of the digestive system, increasing the speed of intestinal transit. This complex mix of dietary compounds is considered a functional food component because it contributes significantly to human health, reducing the risk of chronic diseases [165,166,167]. The consumption of WEFs rich in dietary fibres and non-digestible polysaccharides offers numerous health benefits. So, the wild fruits of the strawberry tree and *A. marmelos* are excellent sources of dietary fibres, vitamins, and minerals, with a low energy contribution. It represents an interesting nutritional value, which contributes to maintaining good health [167,168,169]. Dietary fibres and polysaccharides of WEFs offer significant health benefits beyond their nutritional value, which has an important potential for use as functional foods.

Polysaccharides are a major component of dietary fibres found in WEFs. Dietary fibres can be classified into soluble and insoluble fibres:

- Soluble Fibres: These include gums, mucilages, and pectins, which can be considered as prebiotics fermented by gut microbiota, aiding carbohydrate and lipid metabolism. Pectins are found in fruits like marula and *Pouteria glomerata*, and they are known for their gelling properties and health benefits, such as improving gut health and reducing cholesterol levels [166,170,171]. They augment dietary volume, facilitating regular bowel movements and averting constipation. Soluble fibres have hypocholesterolemic effects by binding to cholesterol molecules, thus impeding their absorption into the bloodstream [172,173,174]. This reduction in cholesterol intake subsequently diminishes the risk of cardiovascular diseases, including heart attacks and strokes [175]. Dietary fibres can also confer a heightened sense of fullness, curb appetite and aid in effective weight management. Such attributes are especially significant in addressing the global surge in obesity and its associated health concerns [176]. In addition to this, selected types of carbohydrates can also selectively stimulate the immunomodulating reaction, stimulating an increase or decrease in cytokines of importance for our immune responses [177].
- Insoluble Fibres: Comprising cellulose, hemicellulose, lignins, and other indigestible components. These fibres are pivotal in promoting digestive well-being [178,179] and contribute to aiding in digestive health by promoting bowel regularity [172,173,180] and preventing conditions like colonic diverticulosis, colon cancer and constipation [172,173,174].

### 4.9. Fatty Acids

**Figure 6:**
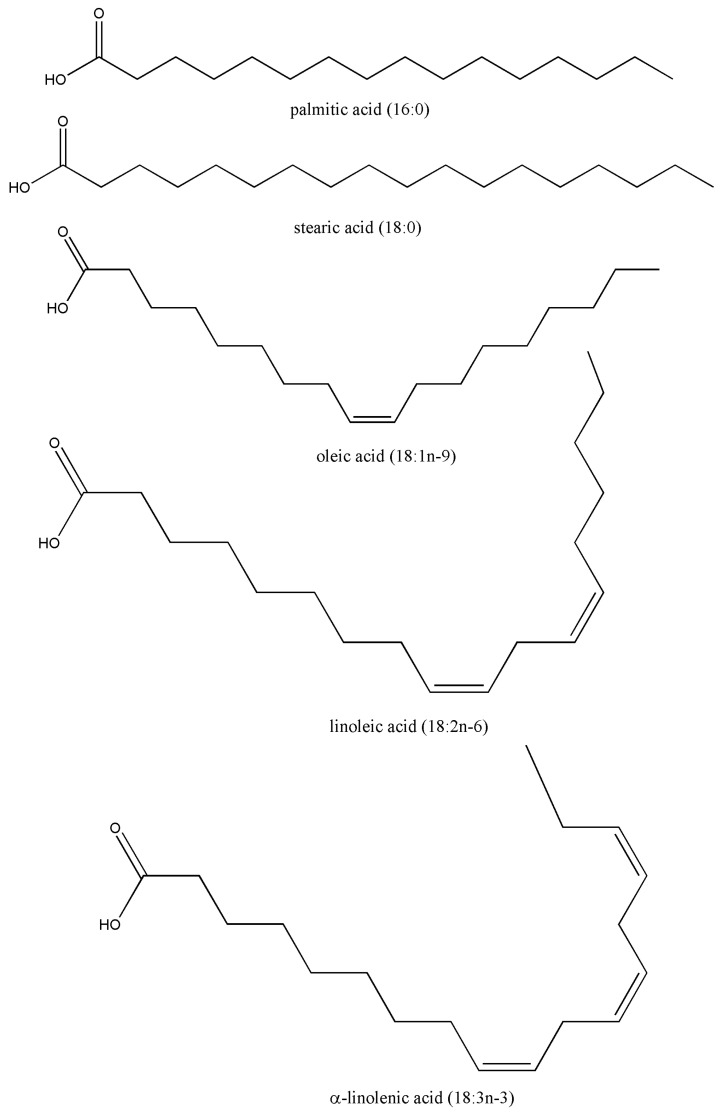
Main fatty acids occurring in Wild Edible Fruits.

Because humans are unable to synthesise essential fatty acids endogenously, their regular intake through diet is indispensable. Although WEFs are generally low in total lipid content, they represent valuable natural sources of nutritionally important fatty acids that contribute to lipid quality rather than quantity in the human diet [181]. In particular, WEFs often exhibit favourable proportions of polyunsaturated and monounsaturated fatty acids, supporting metabolic and cardiovascular health. The main fatty acids occurring in WEFs are shown in Figure 6.

One of the most relevant nutritional features of WEFs is their balanced ratio of *n*-6 to *n*-3 fatty acids. This balance is critical for regulating inflammatory responses, maintaining endothelial function, and reducing the risk of chronic disorders such as cardiovascular disease and metabolic syndrome [182,183]. Many species contain appreciable levels of linoleic acid (LA, 18:2*n*-6) and α-linolenic acid (ALA, 18:3*n*-3), frequently accompanied by oleic acid (OA, 18:1*n*-9). Additionally, saturated fatty acids, such as capric (CaA, 10:0), lauric (LaA, 12:0), palmitic (PA, 16:0), and stearic (SA, 18:0) acids, are found in WEFs in varying proportions.

As summarised in Table 9, the fatty acid profiles of WEFs vary considerably among taxa and geographical origins. Fruits such as *A. unedo*, *R. idaeus*, *R. canina*, and *R. ulmifolius* are characterised by high proportions of LA and ALA, resulting in lipid profiles particularly suited for supporting anti-inflammatory and cardiovascular functions [95,184]. In contrast, species such as *M. communis* and *Z. jujuba* show dominance of OA, a monounsaturated fatty acid associated with improved lipid metabolism and oxidative stability [185,186].

Despite their modest fat content (generally below 5% fresh weight), the qualitative composition of fatty acids in WEFs enhances their nutritional value [187]. When combined with the high levels of antioxidants and other bioactive compounds commonly found in wild fruits, these lipid profiles provide synergistic protection against oxidative stress and inflammation [188].

**Table 9:** Main fatty acids of the pulp of selected Wild Edible Fruits.

Family	Wild Edible Fruit	Main FAs (FA% of Total FA Area)	Total Lipids/Fatty Acids (% fw)	Region	Reference
Apocynaceae	*Landolphia kirkii*	PA 59, SA 42	-	Eastern and Southern Africa	[189]
Anacardiaceae	*Sclerocarya birrea*	PA 67, SA 9, OA 19	-	Tropical Africa	[189]
Ebenaceae	*Diospyros blancoi*	PA 62, SA 25, OA 9	-	Philippines	[189]
Elaeagnaceae	*Hippophaë rhamnoides*	POA 48, PA 29, OA 7, LA 11	-	Europe to central Asia	[185]
Ericaceae	*Arbutus unedo*	PA 11, SA 4, OA 25, LA 24, ALA 31	1.4	Mediterranean	[95]
Moraceae	*Ficus drupacea*	PA 46, SA 3, OA 7, LA 29, ALA 12	0.92	South China to Tropical Asia and N. Queensland	[190]
	*Ficus exasperate*	PA 7, SA 9, OA9, LA 54, ALA 2	4.28	Tropical Africa	[191]
Myrtaceae	*Myrtus comunis*	LaA 4 PA 16, OA 64, LA 13	-	Mediterranean and Southwest Europe	[185]
Rhamnaceae	*Ziziphus jujube*	CaA 47, LaA 15, PA 6, LA 2	1.1	China, feral in temperate worldwide areas	[186]
Rosaceae	*Crataegus monogyna*	PA 30, SA 4, OA 11, LA 11, ALA 16, LgA 13	1.2	Europe and North Africa	[95]
	*Prunus spinose*	PA 23, SA 14, OA 11, LA 14, ALA 11, ArA 13	2.0	Europe, Western Asia, and Northwest Africa	[183]
	*Rosa canina*	PA 2, SA 14, OA 14, LA 40, ALA 26	0.7	Northern Hemisphere	[192]
	*Rosa* species (Polish)	PA 2–4, SA 1–2, OA 14–20, LA 44–56, ALA 19–31	6–12	Northern Hemisphere	[193]
	*Rubus idaeaus*	PA 5–9, OA 5–9, LA 42–53, ALA 18–24	0.5	Europe and Northern Asia	[184]
	*Rubus ulmifolius*	PA 7, SA 3, OA 23, LA 48, ALA 13	1.4	Western Europe	[95]

### 4.10. Toxics and Antinutrients

The utilisation of WEFs contributes significantly to dietary diversity and food security in resource-constrained regions [121]; however, the consumption of these species is complicated by the presence of secondary metabolites that function as natural defence mechanisms [194]. These compounds, ranging from acute toxins to antinutrients that inhibit mineral absorption, necessitate a rigorous understanding of botanical toxicology to prevent adverse physiological effects [195]. The safety profile of WEFs is not inherent but is often contingent upon the stage of maturity and the specific processing methods employed to neutralise bioactive compounds [196,197].

Table 10 presents an assessment of toxic and antinutrient compounds found in selected WEFs, correlating specific chemical constituents with their potential adverse biological actions.

A critical analysis of these phytochemical profiles reveals a dual nature in wild edible plants. While they are vital reservoirs of micronutrients, the chemical defence mechanisms they possess pose significant risks. The presence of acute toxins, such as cyanogenic glycosides in *S. nigra* and glycoalkaloids in *S. nigrum*, represents the most immediate barrier to safe consumption [196,203]. The liberation of hydrogen cyanide from glycosides is a potent enzymatic reaction that can lead to rapid respiratory failure. Furthermore, toxicity is often dynamic; the degradation of solanine during the ripening of *Solanum* species illustrates that safety is temporally dependent [196]. While acute toxins necessitate immediate caution, the prevalence of antinutrients like phytates and oxalates in species such as *R. natalensis* and *Z. spina-christi* poses a chronic threat [195,199]. High concentrations of these compounds can chelate essential minerals, rendering them bio-unavailable. For populations relying on these fruits to combat malnutrition, this creates a counterproductive cycle where the food source inhibits the absorption of the very nutrients it is meant to provide.

To mitigate these risks, specific detoxification protocols are required to render these fruits safe for human consumption. Thermal processing is the primary method for neutralising heat-labile toxins. For *S. nigra*, boiling the fruit for a minimum of 15 to 20 min is necessary to denature the enzymes responsible for releasing cyanide from sambunigrin [203]. Similarly, while ripening reduces solanine in *S. nigrum*, boiling serves as a secondary safety measure, as solanine is water-soluble and leaches into the cooking medium, which must then be discarded [196]. To address antinutrients such as phytates and oxalates found in *Ziziphus* and *Rhus* species, soaking and fermentation are the most effective protocols. Soaking seeds or fruits in water for 12 to 24 h activates endogenous phytases, which break down phytic acid, while fermentation lowers the pH, optimising the conditions for enzymatic degradation of phytates [197]. For high-saponin fruits like *B. aegyptiaca*, prolonged soaking followed by thorough washing and boiling can reduce saponin content, as these compounds are water-soluble and foam out during the boiling process [195,204]. Ultimately, the integration of these processing techniques is essential to transform these wild species from potential toxicological hazards into viable nutritional resources.

## 5. Bioactivity of Wild Edible Fruits

Most biological activities discussed in this review—including antioxidant, antiproliferative, and antimicrobial effects—derive predominantly from in vitro and cell-based models, which represent the lowest tier in the hierarchy of biomedical evidence. Animal studies provide intermediate support, but the well-designed human clinical trials still remain limited. Therefore, the reported bioactivities should be interpreted as exploratory findings requiring confirmation through rigorously controlled in vivo and clinical investigations evaluating relevant biomarkers and health outcomes.

### 5.1. Antioxidant Activity

WEFs exhibit pronounced antioxidant activity, largely attributable to their rich and diverse pool of bioactive compounds. Antioxidant substances can inhibit or delay oxidative damage of nucleic acids, lipids, and proteins by ROS [205]. There are antioxidant nutrients such as vitamins C, E, and β-carotene and trace elements (selenium, copper, zinc, and manganese), which act as cofactors in antioxidant enzymes. In recent decades, there has been a growing interest in the antioxidant non-nutrients, which are biologically active secondary metabolites of plants [40,41]. These fruits are notable sources of phenolic compounds, flavonoids, vitamins (particularly vitamin C and tocopherols), and other phytochemicals capable of neutralising reactive oxygen species and limiting oxidative damage. Through these mechanisms, WEFs may contribute to the prevention or mitigation of chronic disorders such as cardiovascular diseases, cancer, and neurodegenerative conditions [42,95,206,207].

Consistent positive correlations between total phenolic and flavonoid contents and antioxidant capacity confirm that phenolics are major contributors to the redox-modulating properties of WEFs [43,208,209], although synergistic interactions among multiple constituents—including organic acids, carotenoids, and vitamins—often enhance their overall efficacy.

The antioxidant capacity of WEFs is frequently correlated with their total phenolic and flavonoid content. These interactions help explain why several wild fruits display antioxidant activities comparable to, or exceeding, those of widely consumed cultivated fruits [95,210]. Many wild fruits, such as marula and those from the Amazon and Atlantic Forest biomes, are rich in antioxidants, which help combat oxidative stress and reduce the risk of chronic diseases [166,211]. Phenolic antioxidants act through electron and hydrogen donation, metal chelation, and scavenging of reactive oxygen species, and their activity is commonly evaluated using DPPH^•^, ABTS^•+^, and FRAP assays [42,82,206,212].

Owing to their strong antioxidant potential, WEFs have attracted increasing attention as functional foods and as natural ingredients for nutraceutical and dietary supplement development [42,95,213]. Their traditional use in ethnomedicine—particularly for managing inflammatory conditions, infections, and chronic ailments—further supports their biological relevance and therapeutic promise [19,200,214].

Table 11 summarises representative examples of WEFs with notable antioxidant activity, highlighting the diversity of bioactive compounds involved and the range of analytical assays used to assess their effects. Species such as *C. axillaris* [172], *G. lanceifolia* [176], *P. emblica* [31], and *C. phaea* [177] stand out for their strong radical scavenging capacities and high concentrations of phenolics and vitamins, reinforcing the nutritional and functional value of wild fruits.

Although antioxidant activity of WEF pulps is frequently reported using chemical assays such as DPPH^•^, ABTS^•+^, and FRAP, these methods assess radical-scavenging capacity under controlled in vitro conditions, but they do not directly predict physiological efficacy in humans [200,216]. While valuable for preliminary screening and comparative purposes, such assays do not account for bioavailability, metabolic transformation, tissue distribution, or interactions within complex biological systems.

### 5.2. Antimicrobial and Antifungal Effects

Accumulating evidence indicates that WEFs possess relevant antimicrobial and antifungal activities, positioning them as promising sources of natural agents for controlling pathogenic microorganisms [19,69,214]. These properties are of particular interest in the context of increasing antimicrobial resistance and the growing demand for plant-derived alternatives to synthetic preservatives and therapeutics.

The antimicrobial efficacy of WEFs is largely attributed to their rich content of bioactive compounds, including phenolic acids, flavonoids, anthocyanins, terpenoids, and essential oils. These compounds act through multiple mechanisms, such as disruption of microbial cell membranes, inhibition of enzyme activity, interference with nucleic acid synthesis, and prevention of biofilm formation [69,217,218]. In many cases, antimicrobial effects are closely linked to the antioxidant and anti-inflammatory properties of these phytochemicals, which collectively impair microbial survival and virulence [19,219].

In addition to specialised phytochemicals, the overall nutritional matrix of WEFs—including dietary fibres, organic acids, vitamins, and minerals—may enhance antimicrobial and antifungal activity either directly or synergistically by modulating microbial growth conditions [19,220]. This combination of high nutritional value and bioactivity supports the use of wild fruits as multifunctional agents in both food and health applications.

Table 12 summarises representative WEFs exhibiting antimicrobial and/or antifungal activity against a wide spectrum of bacterial and fungal pathogens. Fruits from genera such as *Rubus*, *Vaccinium*, *Syzygium*, *Garcinia*, and *Punica* consistently show strong inhibitory effects against foodborne, opportunistic, and clinically relevant microorganisms. In several cases, fruit extracts demonstrate activity comparable to conventional antimicrobial agents, particularly when concentrated phenolic fractions or peel-derived extracts are used [214,221].

Importantly, antimicrobial potency is often tissue-specific. For species such as *O. ficus-indica*, *A. unedo*, and *C. monogyna*, leaves or cladodes generally exhibit higher antimicrobial activity than the edible fruit pulp, reflecting greater accumulation of tannins and polyphenols. Within the fruit itself, peels frequently represent the most active fraction, as observed in *P. granatum* and *G. gummi-gutta*. From a mechanistic perspective, antifungal activity in the fruits of species belonging to *Vaccinium* and *Rubus* genera is primarily associated with the disruption of fungal cell membranes and inhibition of biofilm formation, rather than direct fungicidal action.

Antimicrobial and antifungal properties of WEFs reinforce their relevance as natural preservatives and for pharmaceutical development. Despite their considerable potential, several challenges limit the broader application of WEFs as antimicrobial and antifungal agents. These include the need for more comprehensive studies addressing compound standardisation, bioavailability, safety, and toxicity, as well as sustainable harvesting practices to prevent overexploitation and habitat degradation [219,222,223]. With appropriate validation and sustainable management, wild fruits may contribute to future strategies aimed at improving food safety and combating microbial resistance.

**Table 12:** Selected examples of the pulp of Wild Edible Fruits with antimicrobial and antifungal activity.

Family	Species	Distribution	Antimicrobial Activity (Effectiveness & Targets)	Antifungal Activity (Effectiveness & Targets)	Reference
Adoxaceae	*Sambucus nigra*	Europe, extending to Western Asia and North Africa	High Activity: Active against respiratory tract pathogens. *Branhamella catarrhalis* *Streptococcus pyogenes*	High Activity: Inhibits *Candida albicans* growth; clinical potential for oral candidiasis management.	[224,225]
Cactaceae	*Opuntia ficus-indica*	Native to Mexico; introduced in Central America, Southern USA, Africa, Asia and Southern Europe	High Activity: Cladodes (pads) show higher activity than fruit pulp, but fruit peel is active. *Salmonella typhi* (3.40 mg/mL MIC) *Helicobacter pylori*	Very High Activity: Strong inhibition of mycelial growth in crop pathogens. *Fusarium oxysporum* *Aspergillus brasiliensis* (Fruit peel extract)	[226,227]
Clusiaceae	*Garcinia gummi-gutta*	Southern India	High Activity: Rind/fruit extracts mediated AgNPs (silver nanoparticles) show enhanced efficacy. *Klebsiella pneumoniae* *E. coli*	High Activity: Potent anti-mucormycotic activity (fighting “Black Fungus”). *Rhizopus arrhizus* *Mucor circinelloides*	[228,229]
Ericaceae	*Arbutus unedo*	Mediterranean area	Moderate Activity: Leaf extracts are significantly more potent than fruits. Fruit extracts showed mild inhibition of *Listeria monocytogenes*.	Moderate Activity: *Candida albicans* inhibition is present but strongly dependent on the arbutin concentration in the extract.	[230,231]
	*Vaccinium myrtillus*	Northern Hemisphere	High Activity: Anthocyanin-rich extracts effectively target Gram-negative bacteria. *Pseudomonas syringae* (MIC: 12.5%) *Escherichia coli*	Moderate Activity: Inhibits growth of *Botrytis cinerea* (Gray mold) and *Candida* spp. primarily through disruption of cell membrane integrity.	[232,233]
Glossuriaceae	*Ribes aureum*	Western U.S.	Moderate to High: Phenolic content correlates directly with inhibition of foodborne pathogens. *Listeria monocytogenes* *Bacillus cereus*	High Activity: “Corona” cultivar extracts suppressed yeasts in food processing environments, outperforming other *Ribes* species.	[234,235]
Lythraceae	*Punica granatum*	Mediterranean Europe, Africa, and Asia	Very High Activity: Peel extracts are among the most potent natural antimicrobials. Broad-spectrum bactericidal (Gram+ and Gram-).	High Activity: Nobiletin and tannins in the peel inhibit *Rhizopus stolonifer* and *Colletotrichum* (post-harvest decay fungi).	[236,237,238]
Malvaceae	*Adansonia digitate*	Central and Southern Africa	Considerable Activity: Methanolic pulp extracts are comparable to standard antibiotics for specific strains. *Enterococcus faecalis* (High sensitivity) *S. aureus* (Leaf extracts show higher potency than pulp)	Low/Specific Activity: Limited broad-spectrum antifungal data; activity is localised to specific environmental moulds affecting the fruit itself.	[239,240,241]
Moraceae	*Artocarpus lacucha*	N. & E. India to S. Central China and Indo-China	Considerable Activity: Bark and fruit extracts inhibit enteric bacteria. *E. coli* *Shigella dysenteriae*	Low Activity: Antifungal activity is minimal compared to its antibacterial and anti-parasitic (anthelmintic) properties.	[242]
Mytaceae	*Syzygium cumini*	Indian subcontinent, Southeast Asia, and parts of Australia	Very High Activity: Fruit juice and seed extracts are bactericidal. *Salmonella typhimurium* *Staphylococcus aureus* (Strong inhibition by seed extract)	Moderate Activity: Extracts effectively inhibit *Candida* biofilm formation and dermatophytic fungi (*Trichophyton* spp.).	[243,244,245,246]
Rhamnaceae	*Ziziphus jujuba*	N. & E. China to S. Korea	High Activity: Polysaccharides and free phenolics in the fruit drive activity. *Staphylococcus aureus* *Salmonella enteritidis*	Moderate Activity: Seed oil inhibits *Aspergillus niger* and *Penicillium* spp.; fruit pulp has lower antifungal efficacy.	[247,248]
Rosaceae	*Cornus mas*	Central to southern Europe and eastwards to Asia Minor	High Activity: Methanol fruit extracts are more effective than water extracts. *Pseudomonas aeruginosa* (Strongest effect) *S. aureus* (0.156 mg/mL MIC)	Specific Activity: Only methanol extracts showed activity against *Candida albicans*; water extracts were inactive.	[249,250]
	*Crataegus monogyna*	Europe and western Asia	Moderate Activity: Leaves generally show lower MICs (better activity) than fruits. *S. aureus* (12 mm zone for leaf extract)	High Activity: Extracts (particularly seed/leaf) effective against dermatophytes (*Trichophyton* spp.).	[251,252]
	*Rosa canina*	Europe, northwest Africa, and western Asia	High Activity: Fruit extracts (especially seeds) show strong inhibition against multidrug-resistant strains. *S. aureus* (MRSA strains) *Acinetobacter baumannii*	High Activity: Significant inhibition zones against non-albicans *Candida*. *Candida glabrata* (20 mm) *Candida tropicalis* (16 mm)	[253,254]
	*Rubus idaeus*	Europe and northern Asia	High Activity: Aqueous fruit extracts significantly inhibit oral and biofilm-forming bacteria. *Streptococcus mutans* (26 mm zone) *Pseudomonas aeruginosa* (Biofilm disruption)	High Activity: Effective against oral and opportunistic fungi. *Candida albicans* (24 mm zone) *Aspergillus niger*	[255,256]
	*Rubus ulmifolius*	Western Europe	High Activity: Leaf extracts show superior activity to fruit; fruit is active against skin pathogens. *Propionibacterium acnes* *S. epidermidis*	High Activity: Significant inhibition of *Penicillium* spp. and *Aspergillus fumigatus* (26.8 mm zone for leaf extract).	[257,258]

### 5.3. Anti-Inflammatory and Analgesic Actions

WEFs are rich sources of bioactive compounds with well-documented anti-inflammatory and analgesic properties. Phytochemical classes, such as anthocyanins, flavonoids, phenolic acids, triterpenoids, and selected vitamins, play central roles in modulating inflammatory cascades and pain perception, thereby contributing to the traditional and emerging therapeutic relevance of these fruits.

#### 5.3.1. Anti-Inflammatory Mechanisms

Multiple mechanisms underlie the anti-inflammatory effects of WEFs. Experimental studies have indicated that fruit-derived bioactives can regulate the production of key inflammatory mediators, including NO, interleukins (e.g., IL-1β, IL-6), TNF-α, and IFN-γ [259]. Suppression of these cytokines attenuates leukocyte recruitment, vascular permeability, and tissue damage associated with acute and chronic inflammation [260].

In parallel, several phytochemicals—particularly phenolics and triterpenoids—directly inhibit pro-inflammatory enzymes and signalling pathways. These include COX-2, PLA2, and NF-κB, leading to reduced synthesis of PGE2 and other inflammatory mediators [259,261]. Such molecular actions are reflected in the targeted therapeutic applications summarised in Table 12, where many WEFs exhibit pathway-specific modulation of inflammation.

#### 5.3.2. Antioxidant–Inflammation Crosstalk

The anti-inflammatory effects of WEFs are closely linked to their antioxidant capacity. Oxidative stress is a key driver of inflammatory signalling, and the ability of fruit-derived antioxidants to scavenge ROS contributes indirectly to inflammation control [69,96,261]. Anthocyanins and flavonoids exert dual antioxidant and anti-inflammatory actions, reinforcing the interconnected nature of these biological effects [19,262].

#### 5.3.3. Analgesic Effects and Pain Modulation

Beyond inflammation control, several WEFs demonstrate direct analgesic activity through modulation of pain pathways. Hydroethanolic extracts of *Byrsonima cydoniifolia* fruits have been shown to reduce oedema and polymorphonuclear leukocyte migration, key contributors to inflammatory pain [263]. Similarly, aqueous extracts of *Terminalia chebula* decrease nociceptive responses in formalin-induced pain models, suggesting modulation of peripheral pain receptors [264].

Essential oils and hydroalcoholic extracts from fruits such as *Heracleum persicum* further demonstrate analgesic potential by significantly reducing acetic acid-induced abdominal constrictions and attenuating both neurogenic and inflammatory pain phases in animal models [265]. These effects are consistent with the receptor- and enzyme-level interactions reported for several species in Table 13, including modulation of TRP channels, opioid receptors, and central pain signalling pathways.

#### 5.3.4. Integration of Phytochemicals and Nutrients

The therapeutic effects of WEFs arise from the combined action of multiple compounds (Table 13). Phenolics and triterpenoids often exhibit stronger anti-inflammatory potency than other phytochemicals due to their ability to suppress pro-inflammatory gene expression and mediator release [259,261]. Vitamins such as ascorbic acid and tocopherols, together with minerals including potassium and magnesium, further support anti-inflammatory and antioxidant defences by stabilising cellular redox balance and modulating immune responses [96,220,266].

**Table 13:** Biochemical actions of the pulp of selected Wild Edible Fruits related to inflammation and Therapeutic Application.

Therapeutic Focus	Species (Family)	Bio-Pathway (Inflammation/Pain)	Key Compounds	Reference
Joint & Arthritis (*Rheumatism*, *Swelling*)	*Aristotelia chilensis* (Elaeocarpaceae)	Selective COX-2 inhibition (Gastric safe)	Delphinidins	[267]
	*Berberis vulgaris* (Berberidaceae)	Downregulates IL-1β gene expression	Berberine	[268]
	*Rubus idaeus*	Blocks NF-κB nuclear translocation	Ellagic acid	[269]
	*Opuntia ficus-indica* (Cactaceae)	Inhibits PLA2 and COX-2 enzymes	Betalains	[270]
	*Myrtus communis* (Myrtaceae)	High-affinity COX-2 binding (Docking)	Myrtle essential oils	[271]
	*Rosa canina* (Rosaceae)	Relieves arthritis; inhibits pain sensation	Galactolipids	[253,272]
Neuropathic & Central Pain (*Nerve Damage*, *Migraine*)	*Solanum nigrum* (Solanaceae)	Modulates opioid receptors (Central pain)	Solamargine	[273]
	*Morus nigra* (Moraceae)	Desensitizes TRPA1/TRPV1 pain receptors	Kuwanon G, Rutin	[274]
Respiratory & Immune (*Infections*, *Asthma*)	*Sambucus nigra* (Adoxaceae)	Modulates cytokine production (IL-6)	Cyanidin-3-glucoside	[275,276]
	*Hippophae rhamnoides* (Elaeagnaceae)	p38 MAPK pathway inhibition	Isorhamnetin	[277]
	*Lonicera caerulea* (Caprifoliaceae)	Suppresses JAK/STAT/NF-κB axis	Iridoids	[278]
Gut & Urinary Health (*Colitis*, *UTI*)	*Vaccinium macrocarpon* (Ericaceae)	Blocks TLR4-mediated signaling	Proanthocyanidins	[279]
	*Eugenia uniflora* (Myrtaceae)	Stops neutrophil migration to injury	Sesquiterpenes	[280]
	*Cornus mas* (Cornaceae)	Reduces C-reactive protein levels	Loganic acid	[281]
Skin & Systemic Health (*Oedema*, *UV Damage*)	*Empetrum nigrum* (Ericaceae)	Blocks UVB-induced ROS generation	Anthocyanins	[282]
	*Prunus spinosa* (Rosaceae)	Inhibits Elastase and TNF-α secretion	Procyanidin B2	[283,284]
	*Physalis peruviana* (Solanaceae)	Downregulates TPA-induced oedema	Withanolides	[285,286]
	*Fragaria vesca* (Rosaceae)	Scavenges NO; Inhibits proteasome	Agrimoniin	[287,288]

### 5.4. Cytotoxic and Anticancer Potential

WEFs have emerged as promising sources of bioactive compounds with cytotoxic, antiproliferative, and chemopreventive properties. A growing body of in vitro evidence demonstrates that extracts from WEFs exert significant antitumor activity against a wide spectrum of human cancer cell lines, supporting their potential role in cancer prevention and complementary therapy [200,289]. These effects are primarily attributed to diverse phytochemical classes—including polyphenols, flavonoids, anthocyanins, iridoids, isothiocyanates, and ellagitannins—which act through multiple molecular mechanisms relevant to carcinogenesis [290,291].

#### 5.4.1. Antiproliferative Activity and Cell Line Sensitivity

As summarized in Table 13, WEF extracts display variable but often potent cytotoxic effects across commonly used cancer models, including breast (MCF-7, T47D), colon (HT-29, HCT-116, Caco-2), liver (HepG2), lung (A549, NCI-H460, H1299), prostate (PC-3, LNCaP), ovarian (SKOV3, OVCAR-3), cervical (HeLa), and leukemia (HL-60) cell lines. GI_50_ and IC_50_ values of the WEF extracts generally fall within the low to mid µg/mL range, indicating that they could have biologically relevant antiproliferative activity.

Wild berries are particularly well represented among the most active species. *V. myrtillus*, *E. nigrum*, *R. idaeus*, *R. canina*, and *S. nigra* exhibit consistent cytotoxic effects, largely linked to their high anthocyanin and ellagitannin content [6,292]. In several cases, seed or flower extracts (e.g., *R. idaeus* and *P. spinosa*) show substantially lower IC_50_ values than pulp extracts, highlighting the influence of tissue-specific phytochemical distribution.

#### 5.4.2. Mechanisms of Anticancer Action

Mechanistic studies reveal that WEF-derived compounds target multiple hallmarks of cancer. Apoptosis induction is one of the most frequently reported mechanisms, involving mitochondrial membrane depolarisation, caspase activation, PARP cleavage, and increased Bax/Bcl-2 ratios, as observed for *V. myrtillus*, *P. spinosa*, *O. ficus-indica*, and *P. peruviana* (Table 14). Some compounds, such as withanolides from *P. peruviana* and solamargine from *S. nigrum*, exhibit particularly strong pro-apoptotic activity at low concentrations.

Other WEFs interfere with cell cycle progression and oncogenic signalling pathways. Iridoids from Cornus mas suppress STAT3 signalling, while isorhamnetin from *H. rhamnoides* inhibits the PI3K/AKT/mTOR axis, both of which are central to tumour cell survival and proliferation. Inhibition of NF-κB and Wnt/β-catenin signalling has also been reported, notably for *S. cumini*, where gallic acid selectively targets cancer stem cell populations.

Anti-angiogenic and anti-metastatic effects further contribute to the anticancer profile of WEFs. Anthocyanins from *E. nigrum* reduce vascular endothelial growth factor (VEGF) expression, while cyanidin-3-glucoside from *M. nigra* inhibits matrix metalloproteinases (MMP-2 and MMP-9), limiting tumour invasion and metastasis.

#### 5.4.3. Comparative Potency and Extract Dependence

Comparative analysis of IC_50_ and GI_50_ values indicates substantial variability among species and extract types. Ethyl acetate extracts, such as those from pomegranate seeds, often exhibit greater cytotoxic potency than aqueous or hydroalcoholic extracts, reflecting selective enrichment of lipophilic anticancer compounds [310,311]. Moreover, differences between fruit tissues (pulp, seed, peel, or flower) significantly influence observed activity, underscoring the importance of standardised extraction and reporting protocols.

#### 5.4.4. Limitations and Translational Challenges

Despite promising in vitro and in vivo findings, translation of WEF-derived bioactivities into clinical relevance requires cautious interpretation. Many reported effects are based on concentrations that may not be achievable through habitual dietary intake. Moreover, bioavailability constraints—including limited absorption, rapid metabolism, and low systemic persistence of polyphenols and carotenoids—may substantially reduce physiological efficacy [312]. Dose–response relationships are rarely established, and potential interactions with other dietary components remain insufficiently explored. In addition, toxicological evaluations are limited for several species, underscoring the need for safety assessments before large-scale nutraceutical or therapeutic applications [44,292].

Despite the substantial body of literature describing antioxidant, anti-inflammatory, antimicrobial, and antiproliferative activities of WEFs, the evidence base remains predominantly preclinical. Within the studies analyzed in this review, approximately 75–85% correspond to in vitro assays (e.g., DPPH^•^, ABTS^•+^, FRAP, cell-based antioxidant or cytotoxicity models) [67,200,312], while 10–20% involve animal models. In contrast, fewer than 5% of the cited studies represent controlled human intervention trials, and these typically assess surrogate biomarkers of oxidative stress rather than clinically meaningful endpoints. This imbalance highlights a significant gap in the evidence hierarchy. Although in vitro assays are valuable for mechanistic screening, they do not account for bioavailability, metabolism, achievable dietary intake levels, dose–response dynamics, or long-term safety. Therefore, health-related claims regarding WEFs should be interpreted cautiously until substantiated by well-designed randomised clinical trials.

#### 5.4.5. Future Perspectives

Future research should move beyond descriptive bioactivity screening toward integrative approaches that consider realistic intake levels, bioavailability, and dose–response relationships. Human intervention studies are essential to determine effective and safe consumption ranges and to validate mechanistic findings observed in vitro. Standardised protocols for bioaccessibility, pharmacokinetics, and long-term safety evaluation should be prioritised [312]. Additionally, toxicological profiling of lesser-known species is necessary to prevent unintended health risks. Such evidence-based strategies will support responsible incorporation of WEFs into functional foods and nutraceuticals while ensuring consumer safety and regulatory compliance.

### 5.5. Metabolic and Cardiovascular Benefits

WEFs exert a wide range of metabolic and cardiovascular benefits owing to their rich composition of bioactive phytochemicals, vitamins, minerals, and dietary fibres. These benefits extend beyond their well-documented antioxidant and anti-inflammatory properties to include improvements in lipid metabolism, vascular function, blood pressure regulation, and thrombosis prevention. Regular consumption of WEFs may therefore play a meaningful role in the prevention and management of chronic metabolic disorders, particularly cardiovascular diseases.

The cardioprotective effects of WEFs arise from multiple, synergistic mechanisms (Table 15). Antioxidant compounds—such as anthocyanins, flavonoids, and phenolic acids—protect circulating lipoproteins and vascular endothelial cells from oxidative damage, thereby limiting low-density lipoprotein (LDL) oxidation and atherogenesis [313,314,315]. This antioxidant action is closely interconnected with anti-inflammatory effects, as reduced oxidative stress dampens inflammatory signalling pathways that contribute to endothelial dysfunction and development of atherosclerotic plaques [19,69,316].

WEFs also influence lipid metabolism by modulating cholesterol synthesis, absorption, and clearance. Regular intake has been associated with improvements in plasma lipid profiles, including reductions in total cholesterol, LDL cholesterol, and triglycerides, alongside favourable effects on high-density lipoprotein (HDL) levels [313,315,317]. These effects contribute to a lower risk of atherosclerosis and coronary artery disease.

Blood pressure regulation represents another key cardiovascular benefit of WEF consumption. Specific bioactive compounds—together with minerals such as potassium and magnesium—support vascular relaxation, improve endothelial nitric oxide bioavailability, and modulate renin–angiotensin–alosterone system activity, collectively contributing to antihypertensive effects [313,314]. In parallel, several wild fruits inhibit platelet aggregation and reduce thrombogenic potential, lowering the risk of cardiovascular events associated with excessive clot formation [317,318].

Beyond cardiovascular health, WEFs exert beneficial effects on broader metabolic processes. Phenolic-rich fruits have demonstrated anti-diabetic properties by improving insulin sensitivity and glucose homeostasis, while their fibre content supports gastrointestinal health and contributes to body weight regulation, thereby indirectly reducing cardiometabolic risk [19,316]. These multifaceted actions help explain the longstanding use of wild fruits in traditional medicine for managing cardiovascular and metabolic disorders.

**Table 15:** Metabolic and cardiovascular benefits of the consumption of the pulp of selected Wild Edible Fruits.

Benefits	Description	Reference
Antioxidant Activity	Wild fruits contain anthocyanins and flavonoids that scavenge free radicals, reducing oxidative stress.	[19,69,316]
Anti-inflammatory Effects	Bioactive compounds in wild fruits help reduce inflammation, which is crucial for preventing chronic diseases.	[19,69,316]
Cardiovascular Protection	Wild fruits help in protecting vascular endothelial function, regulating lipid metabolism, and modulating blood pressure.	[313,314,318]
Lipid Profile Improvement	Consumption of wild fruits can lower plasma lipid levels, reducing the risk of atherosclerosis.	[313,315,317]
Blood Pressure Regulation	Certain wild fruits, like kiwifruit, have been shown to lower blood pressure.	[313,314,318]
Platelet Function Inhibition	Wild fruits inhibit platelet aggregation, reducing the risk of thrombosis.	[313,317]
Anti-diabetic Effects	Phenolics in wild fruits improve metabolic health and help manage diabetes.	[316]
Anti-obesity Effects	Bioactive compounds in wild fruits help in weight management by reducing obesity-related markers.	[316]
Gastrointestinal Health	Dietary fibers in wild fruits aid in digestion and prevent gastrointestinal disorders.	[19]
Traditional Medicine Uses	Wild fruits are used in traditional medicine to treat various ailments, including cardiovascular diseases.	[19]

Future research should prioritise the transition from descriptive phytochemical profiling and in vitro screening toward rigorously designed human intervention studies. Standardised fruit matrices or well-characterized extracts with defined phytochemical composition should be employed to enable reproducibility and meaningful dose–response evaluation. Particular attention must be given to bioavailability, metabolic transformation, and realistic dietary intake levels, as many bioactive effects reported in vitro occur at concentrations unlikely to be achieved through habitual consumption. Moreover, long-term safety and potential toxicological interactions require systematic assessment. Integrating pharmacokinetic studies with randomised controlled trials using clinically relevant endpoints—rather than surrogate antioxidant markers alone—will be essential to establish translational validity and substantiate evidence-based functional or nutraceutical applications of wild edible fruits.

### 5.6. Neuroprotective and Other Health-Promoting Properties

Common disorders of the nervous system are progressively produced with ageing, including memory decrease, motor incoordination, loss of reflexes and mood, emotional stress, anxiety, and depressive states; these disorders are the first manifestations that evolve towards Alzheimer’s or Parkinson’s diseases. A wide array of bioactive compounds present in WEFs confers significant neuroprotective and broader health-promoting effects. Through their capacity to attenuate oxidative stress and neuroinflammation, regulate neurotransmission, and promote neuronal survival and plasticity, these fruits hold promise for the prevention and management of neurodegenerative disorders. While much of the current evidence is derived from in vitro and preclinical studies, the consistency of observed effects highlights the need for further translational research and well-designed clinical trials.

Table 16 summarises the principal neuroprotective compounds identified in WEFs, with particular emphasis on berries, and highlights their key biological actions. Polyphenols and flavonoids—abundant in many wild fruits and berries—are central to the neuroprotective potential of WEFs. These compounds exert strong antioxidant and anti-inflammatory effects that counteract key pathological drivers of neurodegeneration, including reactive oxygen species accumulation, microglial activation, and cytokine overproduction [45,312,319,320]. In addition to limiting neuronal damage, several flavonoids have been shown to modulate signalling pathways involved in neurogenesis, synaptic plasticity, and neurotransmitter regulation, thereby supporting cognitive function [45,312,319,320,321,322,323]. Among these phytochemicals, anthocyanins—particularly abundant in wild berries—have attracted considerable attention. Experimental studies demonstrate that anthocyanins enhance neuroplasticity, improve neuronal communication, and protect brain tissue from oxidative and inflammatory injury [19,69,312,324]. Their ability to cross the blood–brain barrier further strengthens their relevance as dietary neuroprotective agents. Other phenolic constituents, including tannins, catechins, quercetin, kaempferol, and caffeic acid derivatives, contribute synergistically to the neuroprotective effects of WEFs. These compounds reinforce antioxidant defences, suppress pro-inflammatory mediators, and help maintain redox balance in neural tissues [45,321,325]. Certain species exhibit particularly distinctive profiles; for example, *Corema album* berries contain 5-O-caffeoylquinic acid, which has shown notable neuroprotective activity against oxidative stress in vitro [325]

Beyond polyphenols, other classes of bioactives contribute to the neurological benefits of WEFs. Terpenoids and essential oils exhibit antioxidant and anti-inflammatory activities that may support neuronal integrity, while triterpenoids such as oleanolic acid further modulate inflammatory signaling [45,319]. In addition, vitamins—particularly vitamin C—play an essential role in protecting neurons from oxidative damage and supporting normal brain function. Wild fruits such as wild orange and Indian coffee plum are notable sources of this micronutrient [220]. These findings underscore the potential of WEFs as functional foods and nutraceuticals that support brain health while also contributing to systemic antioxidant and anti-inflammatory defences.

**Table 16:** Neuroprotective compounds contained in the pulp of selected Wild Edible Fruits, in particular in berries.

Bioactive Compounds	Neuroprotective Properties	Sources	Reference
Polyphenols	Antioxidant, anti-inflammatory, and neurogenesis stimulation	Various wild fruits and berries	[19,45,312,319,320,321,322,323,325]
Flavonoids	Antioxidant, anti-inflammatory, neurogenesis stimulation, neurotransmitter regulation	Various wild fruits and berries	[19,45,69,312,319,321,323,324,325]
Anthocyanins	Antioxidant, anti-inflammatory, neuroplasticity enhancement	Berries, wild fruits	[19,69,312,321,323,324]
Tannins	Antioxidant, anti-inflammatory	Various wild fruits and berries	[45,312,321]
Caffeic Acid	Antioxidant, anti-inflammatory	Berries	[321,325]
Catechin	Antioxidant, anti-inflammatory	Berries	[321]
Quercetin	Antioxidant, anti-inflammatory, neuroplasticity enhancement	Berries	[321]
Kaempferol	Antioxidant, anti-inflammatory	Berries	[321]
Oleanolic Acid	Antioxidant, anti-inflammatory	Various wild fruits	[45]
5-O-Caffeoylquinic Acid	Antioxidant, neuroprotection against oxidative stress	*Corema album* berries	[325]
Phenolic Acids	Antioxidant, anti-inflammatory, neurogenesis stimulation	Various wild fruits	[319,322]
Terpenoids	Antioxidant, anti-inflammatory	Various wild fruits	[319]
Vitamin C	Antioxidant, neuroprotection against oxidative stress	Wild orange, Indian coffee plum	[220]
Essential Oils	Antioxidant, anti-inflammatory	Various wild fruits	[45]

### 5.7. Bioavailability, Metabolism, and Gut Microbiota Interactions

WEFs, particularly species within the *Vaccinium*, *Rubus*, and *Prunus* genera, are increasingly valued for their exceptional phytochemical profiles, which often exceed those of cultivated varieties in polyphenol diversity and concentration [326]. While these bioactive compounds—specifically anthocyanins, ellagitannins, and proanthocyanidins—offer significant antioxidant and anti-inflammatory potential, their systemic health benefits are strictly governed by bioavailability and metabolic transformation.

Contrary to direct gastric absorption, approximately 90–95% of dietary polyphenols escape digestion in the small intestine and reach the colon intact [327]. Here, they undergo extensive biotransformation by the gut microbiota. Commensal bacteria, including *Bifidobacterium* and *Lactobacillus* species, perform critical enzymatic reactions such as deglycosylation and ring fission, converting complex high-molecular-weight polyphenols into smaller, bioavailable metabolites like phenolic acids and urolithins [328].

Crucially, this interaction is bidirectional. Wild fruit polyphenols function as prebiotics, selectively modulating the gut ecosystem by promoting the growth of beneficial bacteria, such as *Akkermansia muciniphila*, while inhibiting pathogenic populations [329]. This symbiotic relationship enhances host metabolic health, suggesting that the therapeutic efficacy of WEFs is fundamentally mediated by the functional capacity of the individual’s gut microbiome [328].

## 6. Nutritional and Functional Applications

### 6.1. Nutrient Density and Nutritional Value

Although there is no specific international legislation regarding functional foods, the use of this concept is widely extended in the population [330,331]. A functional food consists of compounds that are biologically and physiologically active, which provide health benefits beyond basic nutritional capacities [332]. These constituents of the functional foods, generally termed bioactive compounds, are natural and commonly vegetable food components (micronutrients or phytochemicals) that interact with one or more components of the living tissues to provide a wide range of beneficial potential effects [333]. WEFs are increasingly recognised as valuable ingredients for functional foods and beverages due to their nutritional density and high concentrations of health-promoting bioactive compounds [19,334]. Their rich nutritional profile and bioactive compounds make them an important component of a healthy diet and a promising area for further research and development [165,166,167,335].

In many cases, WEFs surpass cultivated fruits in key micronutrients, including vitamin C, tocopherols, carotenoids (provitamin A), and essential minerals [19,335,336]. Alongside these nutrients, they are rich sources of anthocyanins, flavonoids, saponins, and carotenoids, which collectively underpin their functional and preventive health effects [19,69,337].

Table 17 highlights a consistent trend within the context of this review by examining a particular case, given the impossibility of doing so in a generalised way: wild *Prunus* species generally exhibit higher concentrations of bioactive phytochemicals and greater antioxidant capacity than cultivated counterparts. In particular, *P. spinosa* and *P. cerasifera* show markedly elevated levels of total phenolics, anthocyanins, carotenoids, and FRAP antioxidant activity compared with cultivated *P. domestica*. The substantially higher anthocyanin content in wild taxa (e.g., *P. cerasifera* and *P. spinosa*) explains their superior antioxidant performance, supporting the hypothesis that wild fruits retain stronger stress-induced secondary metabolite profiles.

Vitamin C and tocopherol differences are more moderate but still tend to favour wild species. In contrast, mineral composition (K, Ca, Mg, Fe, Zn) shows comparatively smaller variation between wild and cultivated fruits, suggesting that domestication more strongly affects secondary metabolism than primary mineral accumulation. Probably, the mineral content in cultivation soils plays an important role in the mineral composition of WEFs.

Overall, these data reinforce the manuscript’s central argument that WEFs represent valuable reservoirs of bioactive compounds with enhanced functional potential relative to commonly cultivated varieties.

The development of functional foods enriched with antioxidants and nutraceuticals aims to reduce ROS and free radical levels. Species such as *M. esculenta* and wild orange exhibit strong antioxidant activity associated with their elevated contents of phenolic and flavonoid compounds [9,347]. These bioactives contribute not only to antioxidant capacity but also to anti-inflammatory and antimicrobial effects, reinforcing the role of WEFs as functional foods [220,348]. Such properties underscore their potential applications in nutraceutical and pharmaceutical development [6,220].

Importantly, the nutritional and functional benefits of WEFs extend beyond individual health outcomes to broader socioeconomic impacts. For many indigenous and rural communities, wild fruits constitute a reliable source of food and income, particularly in regions with limited access to cultivated crops [4,6,121]. Promoting their sustainable use, consumption, and domestication can strengthen food systems, enhance dietary diversity, and support local livelihoods. Consequently, WEFs offer both immediate nutritional advantages and long-term potential for resilient, sustainable, and culturally grounded food systems [349].

### 6.2. Use in Functional Foods and Beverages

Regular consumption of wild fruits has repeatedly been associated with a reduced risk of chronic diseases, including cardiovascular and metabolic disorders, largely due to their strong antioxidant capacity and complementary anti-inflammatory and antimicrobial properties [19,69,350]. These attributes make WEFs particularly attractive for the development of functional foods aimed at supporting immune function, gut health, and overall metabolic balance [19,69,337].

From a technological perspective, WEFs exhibit considerable versatility in food and beverage formulations. They are widely used in ready-to-serve products such as juices and health drinks, where their high antioxidant activity can be retained during processing and storage. For example, bayberry (*Myrica esculenta*) and yellow Himalayan raspberry (*Rubus ellipticus*) have been successfully incorporated into functional beverages with sustained phenolic stability and sensory acceptability [24]. Fermentation further expands their functional potential: both alcoholic and non-alcoholic fermented products derived from wild fruits benefit from enhanced flavour complexity and increased levels of bioactive metabolites, including organic acids and secondary phenolics [351,352]. The incorporation of wild fruit extracts into products like yoghurt and others has shown to enhance antioxidant and antidiabetic properties without compromising sensory quality, making them suitable for functional food development [353].

In addition to beverages, WEFs are traditionally and increasingly used in jams, syrups, teas, jellies, and other minimally processed foods, leveraging their natural sweetness, colour, and therapeutic properties [337,354]. The enzyme inhibitory and antimicrobial activities observed in some WEFs further strengthen their suitability as functional ingredients and natural preservatives. Selected examples of WEFs and their applications in functional foods and beverages are summarised in Table 18.

### 6.3. Development of Nutraceuticals and Supplements

In addition, WEFs represent a promising and largely untapped resource for the development of nutraceuticals and dietary supplements. Fully realising this potential requires an integrated strategy that combines scientific research, technological innovation, socio-economic development, and supportive policy frameworks.

From a scientific perspective, expanded exploratory research is essential to document the genetic diversity, ecological distribution, and propagation potential of wild fruit species [28,336,360]. These efforts should be coupled with comprehensive phytochemical and nutritional profiling to identify, quantify, and standardise bioactive compounds responsible for health-promoting effects [361,362]. In parallel, bioactivity validation and bioavailability studies are needed to support evidence-based nutraceutical claims. Consumer acceptance and sensory evaluation studies also play a critical role, ensuring that newly developed products align with market expectations and cultural preferences [362].

Conservation of vegetable species and sustainable supply are equally critical. The establishment of field gene banks and the improvement of propagation and domestication techniques can safeguard valuable genetic resources while ensuring consistent raw material availability [28]. Investments in modern nurseries, local processing units, and cold-chain infrastructure—particularly in remote or rural regions—can significantly reduce post-harvest losses and strengthen supply chains for nutraceutical primary production [363].

From a socio-economic standpoint, the commercialisation of WEF-based nutraceuticals offers meaningful opportunities for rural development. Cultivation, harvesting, processing, and value addition can generate employment and stimulate local entrepreneurship, particularly within agroforestry and mixed farming systems [336,360,362]. Species such as *Dysoxylum kutejensis* and *Artocarpus integer* illustrate how wild fruits can simultaneously support household income, nutrition, and ecosystem services [22]. Market differentiation strategies—including geographical indication, cultural branding, and certification schemes—can further enhance product value while recognising and preserving indigenous knowledge systems [363].

Policy support is a cornerstone of successful nutraceutical development. Integrating WEFs into national nutrition, agriculture, and biodiversity programs can accelerate their adoption and commercialisation [28,363]. Incentives for cultivation, improved land tenure security, better market access, and investments in rural infrastructure are particularly important for scaling production and ensuring equitable benefit sharing [22]. At the same time, robust regulatory frameworks are required to ensure product safety, quality, and efficacy, thereby enhancing consumer confidence and competitiveness in national and international nutraceutical markets [364,365].

All stages of nutraceutical development from WEFs should be guided by sustainability principles. Their integration into diversified farming and agroforestry systems promotes agrobiodiversity, ecosystem resilience, and climate adaptation [335]. Moreover, incorporating WEF-based supplements into diets in low- and middle-income regions may help address micronutrient deficiencies and support public health objectives. International cooperation on conservation, research, and regulatory harmonisation can further align local initiatives with global sustainability and nutrition goals [16].

Finally, technological innovation is essential to support these interconnected pillars. The development of low-cost, scalable processing technologies, along with optimised cultivation and harvesting practices, can improve yield stability, reduce production costs, and expand market access [360,363]. Together, these approaches can foster a resilient, inclusive, and sustainable nutraceutical sector centred on the unique nutritional and bioactive potential of WEFs.

## 7. Sustainability, Cultivation and Ethnobotany

### 7.1. Traditional Harvesting Practices

Traditional harvesting practices of WEFs are deeply embedded in the cultural heritage of indigenous and rural communities worldwide. These practices not only sustain local food systems but also contribute to biodiversity conservation, community resilience, and the intergenerational transmission of ecological knowledge.

In many regions, the collection of WEFs extends beyond subsistence and represents a culturally meaningful social activity. In rural Lebanese communities, for example, wild plant gathering is closely tied to seasonal traditions and collective identity; however, modernisation, urbanisation, and changing land-use patterns have contributed to a gradual decline in these practices [366]. Similar challenges are observed in Indonesia’s Bukit Rimbang–Bukit Baling Wildlife Reserve, where traditional knowledge related to wild fruit harvesting is passed from elders to younger generations but is increasingly threatened by agricultural expansion and weakening knowledge transfer mechanisms [367].

Across Asia, several communities continue to preserve sophisticated ethnobotanical knowledge systems. The Tujia ethnic group in China maintains detailed practices governing the harvesting, processing, and consumption of diverse wild edible fruits, reflecting long-standing ecological adaptation [368]. In the Mizoram region of India, WEFs are integral not only to local diets but also to traditional medicinal practices, underscoring the close relationship between food and health in indigenous knowledge systems [369].

Harvesting techniques vary widely across ecological and cultural contexts but are often inherently sustainable. In Central Kalimantan, Indonesia, selective hand-picking methods are employed to preserve fruit quality while minimising damage to trees and surrounding vegetation [22]. In contrast, communities in Uganda’s Teso–Karamoja region commonly rely on gathering fallen fruits, manual plucking, and shallow digging, practices adapted to local environmental conditions and resource availability [370]. Despite their simplicity, these methods often reflect a deep understanding of plant phenology and ecosystem dynamics.

Nevertheless, traditional harvesting practices are increasingly under pressure. In the Garhwal region of Uttarakhand, India, the erosion of indigenous knowledge—driven by rural-to-urban migration and declining interest among younger generations—poses a serious threat to the continuity of these traditions [2]. In Western Ghats, widespread land-use change, particularly the conversion of forested landscapes into agricultural land, has further restricted access to wild fruit resources [371]. Moreover, rising commercial demand for wild fruits in urban markets has led to unsustainable harvests in some regions, undermining long-term resource availability [372].

In response to these challenges, conservation strategies grounded in traditional knowledge are increasingly being promoted. Community-based forest management systems, which integrate indigenous harvesting practices with modern conservation and governance frameworks, have shown promise in supporting sustainable resource use while empowering local communities [37]. Such approaches highlight the critical role of traditional harvesting practices in aligning biodiversity conservation with cultural preservation and sustainable livelihoods.

**Figure 7:**
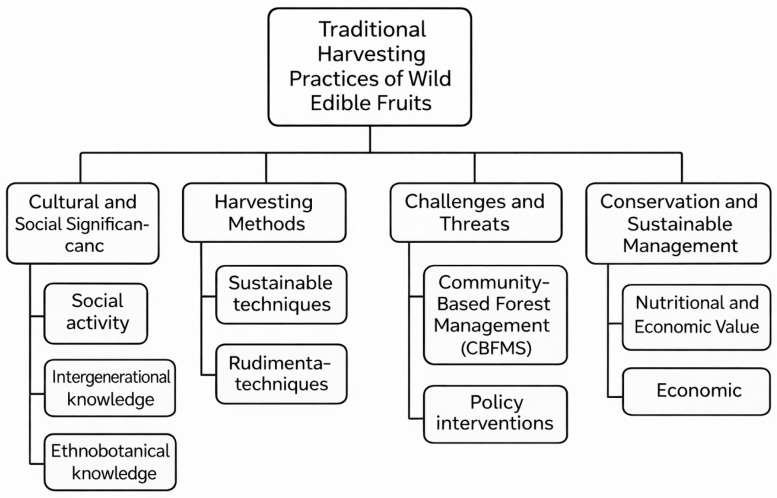
General outline highlighting the main items of the traditional harvesting practices of Wild Edible Fruits.

Figure 7 provides a general overview of the key elements characterizing traditional harvesting practices of WEFs.

### 7.2. Sustainable Management and Domestication

The sustainable management and domestication of WEFs are critical for biodiversity conservation, food security, and economic resilience, particularly within rural and Indigenous communities. Increasing evidence highlights the ecological value of these species, their contribution to local livelihoods, and the opportunities and constraints associated with their long-term sustainable use.

Ethnobotanical surveys across diverse regions reveal the remarkable richness and utility of WEFs. In Central Kalimantan, Indonesia, 61 species belonging to 16 botanical families have been documented [22], while 53 species from 30 genera were recorded in Southeast Aceh [37]. Similarly, the Garhwal region of Uttarakhand, India, hosts at least 69 wild fruit species that contribute to local diets and household incomes [2]. In addition to dietary importance, several species play a key economic and cultural role. For example, *Dracontomelon kutejensis* and *Artocarpus integer* are important income-generating species in Indonesian agroforestry systems [22], while *Blighia sapida* holds both nutritional and cultural significance in Benin [373].

Despite their importance, WEF populations are increasingly threatened. Overharvesting, deforestation, and land-use change have intensified pressure on these natural resources, leading to population decline in several regions [37,374]. The erosion of TEK—driven by rural–urban migration, modernisation, and generational discontinuities—further compromises sustainable management and conservation efforts [2,22].

Effective sustainable use of WEFs requires integrated management strategies that combine indigenous knowledge with modern conservation tools. Community-based forest management and participatory governance frameworks have proven effective in promoting stewardship, regulating harvest intensity, and strengthening local engagement in resource management [37,374]. These approaches allow communities to balance production with conservation while maintaining cultural practices.

Domestication of WEFs often emerges gradually through management in home gardens and agroforestry systems, where wild species are tolerated, protected, or transplanted—a process reflecting early stages of agricultural adaptation [375]. In Benin, traditional practices surrounding *Blighia sapida* illustrate how culturally embedded management can facilitate domestication while preserving genetic diversity [373]. At the same time, sustainable harvesting techniques, such as selective hand-picking and seasonal restrictions, help maintain plant productivity and minimise ecological disturbance [22].

Creating an enabling political environment is essential for scaling up these practices. Secure land tenure streamlined regulatory frameworks, and the formal recognition of agroforestry and community-managed systems can incentivise sustainable use [22]. Improved infrastructure and market access are equally important, as value addition through processing and commercialisation enhances household incomes and creates economic incentives for conservation [374,376,377]. Finally, continued interdisciplinary research—particularly on nutritional composition, medicinal potential, and toxicological safety—is necessary to fully realise the contribution of WEFs to resilient and sustainable food systems [378].

### 7.3. Socio-Economic and Livelihood Impacts

WEFs play a multifaceted role in supporting rural livelihoods, food security, and cultural identity across many regions of the world. However, the long-term benefits derived from WEFs depend critically on sustainable management and effective integration into local development and conservation strategies.

#### 7.3.1. Socio-Economic and Cultural Value

WEFs represent an important source of income for rural and forest-dependent communities, particularly where access to formal markets and agricultural inputs is limited. In the Boundou Region of Senegal, for example, the commercialisation of baobab fruits provides essential cash income and strengthens household food security [379]. Similarly, in Namibia and Zimbabwe, wild fruits are widely harvested and sold to supplement household earnings while also contributing to daily nutrition [21].

In addition, these resources contribute to resilience against food shortages and the preservation of traditional knowledge systems. WEFs are deeply embedded in cultural practices and social identities. In southern Africa, traditional knowledge related to wild fruit harvesting, processing, and use reflects long-standing ecological relationships and cultural heritage [21]. In the South Caucasus, variations in fruit use across age groups highlight the role of WEFs in shaping community traditions and intergenerational knowledge transmission [380].

#### 7.3.2. Nutritional and Medicinal Contributions

WEFs contribute substantially to dietary diversity by providing essential micronutrients and bioactive compounds, particularly in nutritionally vulnerable populations. In the Himalayan region, these fruits are recognised for their high nutraceutical value and health-promoting properties [377,381]. In Arunachal Pradesh (India), WEFs are not only consumed as foods but are also widely used in traditional medicine to treat a range of ailments, underscoring their dual nutritional and therapeutic relevance [20].

#### 7.3.3. Food Security and Sustainable Livelihoods

WEFs play a critical role in enhancing food security, especially in regions characterised by marginal agricultural conditions or seasonal food scarcity. In East Aceh, Indonesia, they constitute a key nutritional and economic resource for rural households [23]. In the Kashmir Himalayas, although their direct economic contribution is relatively modest, wild edible plants and fruits significantly support urban and peri-urban food and nutritional security [382].

Participatory conservation initiatives in the central Himalayas demonstrate that sustainable management of WEFs can simultaneously support biodiversity conservation and livelihood diversification [383,384]. Moreover, value-added processing—such as the extraction of pectin from wild fruits in Odisha (India)—illustrates how WEFs can create alternative income streams and enhance local economic resilience [171].

#### 7.3.4. Environmental Pressures and the Need for Conservation

Despite their socio-economic importance, WEFs face increasing environmental and socio-economic pressures. In Veracruz (Mexico), the land-use change, deforestation, and habitat fragmentation threaten wild fruit populations, highlighting the urgency of targeted conservation measures [385]. In Kyrgyzstan (post-Soviet), some economic transformations have contributed to the degradation of walnut fruit forests, illustrating how broader socio-economic shifts can undermine traditionally managed forest systems [386].

## 8. Challenges, Gaps and Future Directions

### 8.1. Standardisation of Analyses and Validation of Bioactivities

The interpretation of phytochemical composition and antioxidant capacity data across studies is complicated by methodological heterogeneity. Spectrophotometric assays (e.g., Folin–Ciocalteu for total phenolics) provide rapid global estimates but lack compound specificity and may overestimate concentrations due to interference from reducing substances. In contrast, chromatographic techniques, such as HPLC-DAD or LC–MS, offer higher specificity and structural resolution; however, they are more sensitive to extraction conditions, calibration standards, and instrument parameters. Differences in solvent systems, extraction time, sample preparation (fresh vs. dried material), and expression units (fresh weight vs. dry weight; mg GAE vs. mg/kg) further contribute to inter-study variability. Consequently, direct quantitative comparisons between species or regions should be interpreted cautiously unless analytical protocols are harmonized. Greater standardization in extraction procedures, reporting units, and compound identification criteria would substantially enhance comparability and reproducibility in WEF research.

Reliable characterisation of WEFs requires standardised analytical methodologies to ensure comparability across studies. The availability of official methods for determining the different components or characteristics of many food samples facilitates the selection of a method. The AOAC reports validated protocols with the necessary analytical characteristics for the proximate, nutritional, and phytochemical food analyses [387], which provides baseline data on macronutrients, vitamins, and minerals [388,389].

Many different physicochemical techniques, including spectroscopic, chromatographic, electrophoretic, biochemical, immunoassay, and microscopic techniques, have been proposed to analyse WEFs [390]. These instrumental methods have improved the analytical characteristics, and consequently, they are faster and more profitable, with a higher number of samples analysed per unit of time. For method selection, the content of the analysed components and the interfering components must also be considered. Therefore, the analytical characteristics of any method, such as specificity, sensitivity, precision, and accuracy in the analysis, are obviously determining parameters for the choice of the method [391]. The analysis of standard reference material or check samples used as controls is a major consideration for establishing the validation of an analytical method [392].

Instrumental techniques with great possibilities are being used, such as liquid or gas chromatography coupled with different detectors, highlighting mass detectors. Advanced chromatographic techniques—such as HPLC-DAD/MS for phenolics and flavonoids, GC–MS for volatile and lipid-soluble compounds, and HPTLC–UV/Vis for rapid screening—are widely applied to quantify bioactive constituents with high sensitivity and reproducibility [393]. Mineral composition is typically determined by atomic absorption spectrophotometry (AAS) using flame air or nitrous/acetylene or graphite chamber. Other techniques, such as plasma-based atomic absorption (ICP-OES, or ICP-MS) or emission spectrophotometry, have also been developed [387,390,391,392,394].

Despite these advances, methodological heterogeneity in extraction, quantification, and reporting remains a major limitation. Harmonised analytical workflows and standardised reference materials are needed to improve data consistency and reproducibility [395].

A wide range of bioactivities—including antioxidant, anti-inflammatory, antimicrobial, and anticancer effects—has been demonstrated for WEFs through in vitro and in vivo studies [19,69,200]. Antioxidant capacity is commonly evaluated using DPPH^•^, ABTS^•+^, and FRAP assays, while antimicrobial and cytotoxic activities have been confirmed against multiple pathogens and cancer cell lines [396,397]. However, human clinical evidence remains scarce. Future research should prioritise well-designed clinical trials to validate efficacy, establish safe dosage ranges, and clarify the bioavailability and metabolic fate of WEF-derived bioactives.

### 8.2. Bioprospecting, Intellectual Property, and Benefit Sharing

It is evident that WEFs represent valuable reservoirs of bioactive compounds with significant potential for bioprospecting in functional foods, nutraceuticals, and pharmaceutical applications [16,69]. However, rapid loss of ethnobotanical knowledge and inadequate documentation pose risks to equitable intellectual property protection and increase vulnerability to biopiracy [383].

Therefore, protecting indigenous and local knowledge through legal recognition, farmers’ rights, and community-based intellectual property frameworks is essential [20]. Participatory conservation, sustainable harvesting, and domestication of WEFs within agroforestry systems can simultaneously support biodiversity conservation, food security, and rural livelihoods [222,398]. International access-and-benefit-sharing mechanisms, such as those under the Nagoya Protocol, provide an important framework for ensuring ethical and equitable commercialisation [399].

### 8.3. Technological Innovations and Sustainable Valorisation

Green extraction technologies—including ultrasound, microwave, pulsed electric field, and supercritical fluid-assisted methods—improve recovery of bioactive compounds while reducing solvent use, energy consumption, and environmental impact [226,400].

Nanotechnology, particularly nanoencapsulation, offers additional advantages by improving stability, bioavailability, and controlled release of WEF-derived bioactives. Applications in edible coatings, antimicrobial packaging, and nanosensors have shown promise in extending shelf life and reducing post-harvest losses [401,402]. Green-synthesised nanoparticles, such as plant-derived silver nanoparticles, further enhance preservation efficiency while aligning with sustainability goals [403].

### 8.4. Future Research on Wild Edible Fruits

To validate WEFs as reliable food sources, future research must progress from basic identification to rigorous toxicological assessment. The proposed roadmap prioritises high-throughput screening and bioavailability studies to map chemical risks (Phases 1–2), followed by the optimisation of processing techniques like fermentation to neutralise antinutrients (Phase 3). Ultimately, this trajectory aims to domesticate low-toxin genotypes (Phase 4), establishing evidence-based guidelines that transform these underutilised species into safe, scalable solutions for global food security.

**Figure 8:**
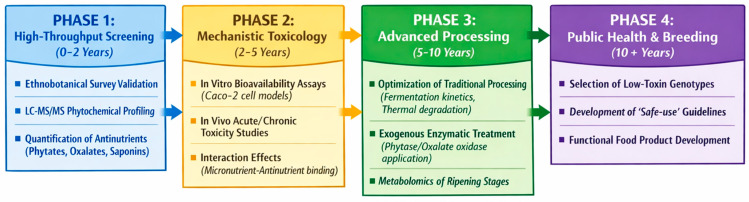
Roadmap for future research on Wild Edible Fruits.

The proposed roadmap (Figure 8) presents a logically staged framework for advancing research on WEFs, integrating phytochemical screening, toxicological validation, processing optimisation, and translational public-health outcomes. The emphasis on early phytochemical profiling and antinutrient quantification is consistent with established knowledge that many wild plant foods contain significant levels of phytates, oxalates, and tannins affecting nutrient utilisation [404]. Likewise, the transition to mechanistic nutritional and bioavailability studies addresses the documented gap between compositional data and effective nutrient contribution of wild foods to diets. The roadmap’s inclusion of traditional processing optimisation and detoxification strategies is well supported, as fermentation and related treatments are known to reduce phytic acid and improve mineral bioaccessibility in plant foods [405,406]. Finally, the breeding and safe-use phase appropriately links biochemical traits to cultivar selection and functional food development, consistent with domestication and conservation pathways for wild food plants [407]. Overall, the roadmap is comprehensive and aligned with current wild food research priorities.

## 9. Concluding Remarks

WEFs represent a highly diverse and underutilised group of plant resources with substantial nutritional, functional, and socio-cultural value. These foods are pivotal for dietary quality, food security, and rural livelihoods, particularly in biodiversity-rich and resource-limited regions. Regional studies underscore their versatility across diverse ecological settings, positioning the pulp—the primary edible tissue—as a rich source of essential nutrients, including vitamins, minerals, polysaccharides, and health-promoting fatty acids, alongside a wide array of bioactive compounds.

From a nutritional perspective, vitamin C stands out as one of the most significant attributes of WEFs. The high ascorbic acid concentrations reported underscore their potential to improve antioxidant intake, immune health, and iron bioavailability. Additionally, the large amounts of polysaccharides and dietary fibers present in these fruits offer a strategic approach to increasing fibre intake in the regular diet. WEFs also constitute a complementary source of beneficial fatty acids and tocols (vitamin E); the latter, though still underexplored, warrants further targeted investigation to fully understand its bioactive contribution. Furthermore, their mineral composition reinforces their status as nutrient-dense resources capable of improving physiological functions in settings where mineral deficiencies remain prevalent.

The functional value of WEFs is primarily driven by phenolics and carotenoids (both nutritional and non-nutritional), which are central to their high antioxidant activity. Regular consumption of these fruits may offer a sustainable strategy to reduce cellular oxidative stress and the risk of degenerative diseases. Furthermore, sterols and triterpenoids constitute a pharmacologically relevant fraction: sterols contribute to lowering blood cholesterol, while other triterpenoids, essential oils, and terpenoids exhibit significant anti-inflammatory, analgesic, and antimicrobial properties.

Collectively, these components have demonstrated a wide range of beneficial effects in in vitro and in vivo studies, including cardiovascular protection (through antihypertensive and antithrombotic pathways), metabolic homeostasis, and neuroprotection. By attenuating neuroinflammation and promoting neuronal survival, WEFs hold promise for the prevention of neurodegenerative disorders.

Traditional harvesting practices and the domestication of WEFs are critical for maintaining biodiversity, food security, and the cultural heritage of indigenous communities. Embedding these species into local food systems and rural development strategies can enhance income generation and the intergenerational transmission of ecological knowledge. While international access-and-benefit-sharing mechanisms provide a framework for ethical commercialisation, challenges related to regulatory approval, safety assessment, and accessibility for small-scale producers remain significant barriers.

Despite this vast potential, several constraints limit the translation of current knowledge into practical applications. Future efforts should prioritise:

1. Standardised Analytical Approaches: To overcome the variability driven by genetic and environmental factors and the lack of harmonised protocols.
2. Clinical Validation: Transitioning from in vitro evidence to well-designed human clinical trials to establish efficacy, safe dosage ranges, and bioavailability.
3. Technological Innovation: Integrating traditional knowledge with modern technologies, such as green extraction and nanoencapsulation, to enhance product stability and marketability.

In conclusion, WEFs offer a unique convergence of nutritional richness, functional bioactivity, and ecological resilience. An integrated approach linking sustainable resource management with scientific research is essential to ensure the long-term sustainability of these fruits and the ecosystem services they provide.

## Figures and Tables

**Table 10:** Toxics and antinutrients in selected Wild Edible Fruits.

Family/Species	Toxic Compounds	Antinutrients	Adverse Actions	Reference
Adoxaceae (*Sambucus nigra*—Elderberry)	Cyanogenic glycosides (Sambunigrin)	Lectins	Ingestion of raw fruit releases hydrogen cyanide, causing nausea, vomiting, and respiratory distress; lectins may cause digestive upset.	[198]
Anacardiaceae (*Rhus natalensis*—Natal Rhus)	None detected (in pulp)	Phytates (High), Oxalates	High phytate levels (1.52 mg/100 g) significantly bind iron and zinc, reducing mineral bioavailability; oxalates may contribute to renal stone formation.	[199]
Balanitaceae (*Balanites aegyptiaca*—Desert Date)	Saponins (Diosgenin)	Tannins, Oxalates	High saponin content can irritate gastric mucosa and cause hemolysis in large quantities; tannins (7.40%) precipitate proteins, reducing digestibility.	[195]
Ebenaceae (*Euclea racemosa*—Bush Guarri)	None detected	Phytates, Tannins	Antinutrients interfere with the absorption of calcium and magnesium; generally safe but excessive consumption limits nutrient uptake.	[199]
Elaeagnaceae (*Elaeagnus caudata*—Silverberry)	None detected	Tannins, Saponins	The presence of tannins can cause astringency and reduce protein digestibility; saponins may cause mild gastrointestinal irritation.	[200]
Moraceae (*Ficus sur*—Cape Fig)	Furocoumarins (potential)	Phytates, Oxalates	Phytates (approx. 1.2 mg/100 g) bind essential minerals; excessive intake may lead to mineral deficiencies in nutrient-poor diets.	[199]
Primulaceae (*Embelia subcoriacea*)	Alkaloids (Embelin)	Tannins, Saponins	Qualitative analysis confirms presence of alkaloids and saponins which may exhibit cytotoxicity or gastrointestinal toxicity in high doses.	[200]
Rhamnaceae (*Ziziphus spina-christi*—Christ’s Thorn)	None detected	Oxalates (Very High)	Extremely high oxalate levels (up to 16.2%) pose a significant risk for kidney stone formation and calcium deficiency if consumed in excess.	[195]
Rosaceae (*Rosa abyssinica*—Abyssinian Rose)	None detected	Saponins (High), Tannins	Highest saponin content (2.12 mg/100 g) among comparable wild fruits; can cause bloating and digestive distress.	[199]
Salicaceae (*Dovyalis abyssinica*—Abyssinian Gooseberry)	None detected	Phytates, Oxalates	Contains moderate levels of phytates and oxalates; inhibits absorption of iron and calcium, but generally lower risk than *Ziziphus*.	[201]
Solanaceae (*Solanum nigrum*—Black Nightshade)	Solanine/Solamargine (Glycoalkaloids)	Saponins	Unripe green berries contain high levels of neurotoxic glycoalkaloids, causing vomiting, diarrhoea, and confusion; ripe berries are generally safe.	[202]
Vitaceae (*Cissus obovata*)	Cardiac glycosides	Alkaloids, Tannins	The presence of cardiac glycosides suggests potential cardiovascular activity; traditional use requires caution due to these bioactive compounds.	[200]

**Table 11:** Selected examples of the pulp of selected Wild Edible Fruits with antioxidant activity.

Family	Fruit	Bioactive Compounds	Antioxidant Activity	Potential Use	Reference
Anacardiaceae	*Choerospondias axillaris*	Phenolic compounds, lycopene, and ascorbic acid	The highest antioxidant activities among the studied fruits	Rich in minerals and phytochemicals, with potential for food and pharmaceutical use	[207]
Clusiaceae	*Garcinia lanceifolia*	Saponin, flavonoids, tannin, alkaloids	High free radical scavenging activity (IC_50_ = 10.37 µg/mL)	Potential resources for ethnomedicine and income generation	[200]
Elaeocarpaceae	*Elaeocarpus serratus*	Phenolic compounds, flavonoids, ascorbic acid	High antioxidant activity, a viable source for functional food applications	Underutilised fruit with significant health benefits	[210]
Moraceae	*Artocarpus gomeziana*	Phenolic compounds, flavonoids	High radical scavenging activity (IC_50_ = 0.19 mg dw)	Potent natural antioxidant source	[206]
Myrtaceae	*Campomanesia phaea*	Phenolic compounds, ascorbic acid, and proanthocyanidins	High antioxidant capacity measured by ABTS^•+^ and ROO^•^ radical scavenging	Higher levels of bioactive compounds than those commonly consumed fruits	[215]
Phyllanthaceae	*Phyllanthus emblica*	Phenolic compounds, ascorbic acid, and anthocyanins	Significant antioxidant activity correlated with phenolic content	Promoted as a natural source of antioxidants/nutraceuticals	[42]
Rosaceae	*Crataegus monogyna*	Tocopherols, vitamin C, and organic acids	High antioxidant activity (β-carotene bleaching and TBARS assays)	High tocopherol content. Used to improve hypertension and poor circulation	[95]
	*Prunus spinose*	Tocopherols, vitamin C, and organic acids	High antioxidant activity (β-carotene bleaching and TBARS assays)	High tocopherol content. antioxidant, anti-inflammatory, laxative, diuretic and stomachic properties	[95]
	*Rubus ulmifolius*	Tocopherols, vitamin C, and organic acids	High antioxidant activity (β-carotene bleaching and TBARS assays)	Functional food ingredient, high tocopherol content	[95]

**Table 14:** Selected examples of anticancer actions of the pulp of selected Wild Edible Fruits.

Family	Species	Cell Line (Organ)	Mechanism of Action	GI_50_/IC_50_/EC_50_	Reference
Adoxaceae	*Sambucus nigra* (Elderberry)	MCF-7 (Breast) HT-29 (Colon)	Membrane agglutination by lectins (SNA); Mitochondrial depolarisation	IC_50_: ~250 µg/mL IC_50_: 0.8 mg/mL	[275,293]
	*Viburnum opulus* (Guelder Rose)	HeLa (Cervix) Caco-2 (Colon)	Chlorogenic acid induces S-phase arrest; Downregulates MMP-9	IC_50_: 55.4 µg/mL IC_50_: 41.2 µg/mL	[294,295]
Cactaceae	*Opuntia ficus-indica* (Prickly Pear)	HepG2 (Liver) OVCAR-3 (Ovary)	Indicaxanthin activates Caspase-3; Induces chromatin condensation	IC_50_: 1.25 mg/mL (Flower) IC_50_: 400 µg/mL (Pulp)	[296,297]
Cornaceae	*Cornus mas* (Cornelian Cherry)	A549 (Lung) SKOV3 (Ovary)	Iridoids block STAT3 signalling; Inhibits colony formation	IC_50_: 147 µg/mL IC_50_: 2.36 mg/mL	[298]
Elaeagnaceae	*Hippophae rhamnoides* (Sea Buckthorn)	HepG2 (Liver) BGC-823 (Gastric)	Isorhamnetin inhibits PI3K/AKT/mTOR pathway	IC_50_: 32.5 µg/mL IC_50_: 45 µg/mL	[299]
Ericaceae	*Vaccinium myrtillus* (Wild Bilberry)	HCT-116 (Colon) HL-60 (Leukaemia)	Delphinidin triggers intrinsic apoptosis pathway; ROS generation	IC_50_: ~50 µg/mL IC_50_: 30 µg/mL	[233,300]
	*Arbutus unedo* (Strawberry Tree)	LNCaP (Prostate) HCT-116 (Colon)	Downregulates Androgen Receptor (AR); DNA fragmentation	IC_50_: 180 µg/mL IC_50_: 500 µg/mL	[301]
	*Empetrum nigrum* (Crowberry)	HT-29 (Colon) HeLa (Cervix)	Anthocyanins reduce VEGF expression (Anti-angiogenesis)	IC_50_: 62.5 µg/mL	[302]
Moraceae	*Morus nigra* (Black Mulberry)	PC-3 (Prostate) MCF-7 (Breast)	Cyanidin-3-glucoside inhibits metalloproteinases (MMP-2/9)	IC_50_: 120 µg/mL GI_50_: 52 µg/mL	[303]
Myrtaceae	*Syzygium cumini* (Jamun)	HCT-116 (Colon) A549 (Lung)	Gallic acid targets CSCs (Cancer Stem Cells); Wnt/β-catenin inhibition	IC_50_: 85.5 µg/mL IC_50_: 60 µg/mL	[304]
Rosaceae	*Rubus idaeus* (Wild Raspberry)	CaCo-2 (Colon) HepG2 (Liver)	Sanguiin H-6 induces G2/M arrest; PARP cleavage	IC_50_: 45 µg/mL (Seed) IC_50_: 728 µg/mL (Pulp)	[305]
	*Prunus spinosa* (Blackthorn/Sloe)	HCT-116 (Colon) U87-MG (Glioblastoma)	Triggers mitochondrial membrane depolarization; Increases Bax/Bcl-2 ratio	IC_50_: 50 µg/mL (Flower) IC_50_: 125 µg/mL (Fruit)	[306]
	*Rosa canina* (Rosehip)	T47D (Breast) NCI-H460 (Lung)	Antiproliferative via p53 activation; Telomerase inhibition	GI_50_: 184 µg/mL IC_50_: 250 µg/mL	[307]
Solanaceae	*Physalis peruviana* (Goldenberry)	H1299 (Lung) MDA-MB-231 (Breast)	4β-Hydroxywithanolide E causes DNA double-strand breaks	IC_50_: 0.71 µg/mL IC_50_: 1.58 µg/mL	[308]
	*Solanum nigrum* (Black Nightshade)	HepG2 (Liver) MCF-7 (Breast)	Solamargine upregulates Fas/FasL (Death receptor pathway)	IC_50_: 20 µg/mL IC_50_: 4.8 µM	[309]

**Table 17:** Comparative phytochemical composition, antioxidant capacity, and mineral content of wild (*P. avium*, *P. spinosa*, *P. cerasifera*) and cultivated (*P. domestica*) *Prunus* fruits (pulp, fresh weight unless otherwise stated).

Parameter	*P. avium* Wild	*P. Spinosa* Wild	*P. cerasifera* Wild	*P. domestica* Cultured	References
Vitamin C mg/100 g fw	7–10	11.17–21.30	3.5–6.6	1.6–2.2	[301,338,339,340]
Total carotenoids mg/kg fw	4.1	10.88	19.6	0.4–1.88	[67,341,342]
Total tocopherols mg/kg fw	1.2	3.14	-	0.5–06	[67,301,338]
Total phenolics mg GAE/100 g fw	237	237–504	49–470	27–54	[67,339,343]
Total anthocyanins mg/kg fw	571	2335	4290	7–108	[67,340,343]
FRAP antioxidant capacity mmol TE/100 g fw	0.73	1.1–1.3	1.12–4.48	0.6–1.28	[67,339,344,345]
Potassium mg/100 g dw	961	1120 ± 140	980 ± 120	870 ± 110	[301,346]
Calcium mg/100 g dw	72	68 ± 12	54 ± 10	43 ± 9	[301,346]
Magnesium mg/100 g dw	55	52 ± 9	46 ± 8	39 ± 7	[301,346]
Iron mg/100 g dw	1.8	4.3 ± 0.7	2.6 ± 0.5	1.8 ± 0.4	[301,346]
Zn mg/100 g dw	0.6	1.1 ± 0.2	0.8 ± 0.2	0.6 ± 0.1	[346]

**Table 18:** Selected examples of Wild Edible Fruits pulps used in functional foods and beverages.

WEFs	Foods and Beverages	Functional Compounds	Reference
*Myrica esculenta*	Health beverages, jams, syrups	Rich in antioxidants, phenolics, flavonoids, and vitamin C	[24,337]
*Rubus ellipticus*	Ready-to-serve health beverages	High in carotenoids and phenolics	[24]
*Prunus spinosa*	Functional food	Rich in phenolics, flavonoids, anthocyanins; antioxidant and enzyme inhibitory activities	[355]
*Docynia indica*	Antioxidant and antimicrobial agents	High phenolic and flavonoid content	[356]
*Viburnum foetens*	Functional food	Rich in polyphenols, essential metals, and antioxidants	[53]
*Mahonia jaunsarensis*	Health-promoting functional foods	Rich in diverse nutrients, minerals, vitamins, and phenolic compounds	[357]
*Azara serrata*	Functional food	Rich in glycosylated anthocyanins and phenolic compounds; enzyme inhibitory activities	[358]
*Ehretia tinifolia*	Functional food	Rich in antioxidants and polyphenols; antiproliferative activities	[359]
*Sideroxylon lanuginosum*	Functional food	High flavonoid content; antioxidant and enzyme inhibitory activities	[359]

## Data Availability

No new data were created or analyzed in this study. Data sharing is not applicable to this article.

## Abbreviations

The following abbreviations are used in this manuscript:

AAS	Atomic absorption spectrometry
A549	Human lung cancer cell line model
ABTS	2,2′-azino-bis-(3-ethylbenzothiazoline-6-sulfonic acid)
ABTS^•+^	ABTS radical cation
AgNPs	Silver nanoparticles
Akt	Protein Kinase B
ALA	Alpha-linolenic acid
AOAC	Association of Official Analytical Collaboration
AR	Androgen receptor
Bax	Bcl-2-associated X protein (Proapoptotic)
Bcl-2	B-cell lymphoma (Antiapoptotic)
BGC-823	Gastric carcinoma cell line model
CaA	Capric acid
CaCo-2	Colorectal adenocarcinoma for cell line model
Caspase	Cysteine-aspartic protease
C-33A	Human uterine cancer cell line model
CBFMS	Community-based forest management
COX2	Cycloooxygenase-2
CSCs	Cancer Stem Cells
CVD	Cardiovascular diseases
DAD	Diode array detector
DPPH	2,2-Diphenyl-1-picrylhydrazyl (stable free radical)
DU145	Human prostate cancer cell line model
DW	Dry weight
EC50	Half (50%) maximal effective concentration
EO	Essential oils
FRAP	Ferric reducing antioxidant power
FW	Fresh weight
G1 phase arrest	Pause in the cell cycle before the S phase, triggered by cell stress, DNA damage, or lack of growth factors
GAE	Gallic acid equivalent
GC	Gas chromatography
GI50	Half (50%) maximal growth inhibitory concentration
H1299	Non-small cell lung carcinoma cell line model
HCT-116	Colorectal carcinoma cell line model
HeLa	Cervical cancer cell line model
HepG2	Liver hepatocellular carcinoma cell line model
HL-60	Promyelocytic leukemia cell line model
HPLC	High-performance liquid chromatography
HPTLC	High-performance thin-layer chromatography
HT-29	Human colorectal adenocarcinoma cell line model
IC50	Half (50%) maximal inhibitory concentration
ICP-MS	Inductively coupled plasma mass spectroscopy
ICP-OES	Inductively coupled plasma optical emission spectroscopy
IFN-γ	Interferon gamma
IL-1β	Interleukin-1 beta
IL-6	Interleukin-6
IL-12	Interleukin-12
JAK	Janus Kinase
LaA	Lauric acid
LA	Linoleic acid
LNCaP:	Prostate carcinoma (Lymph Node) cell line model
MCF-7	Human breast cancer cell line model
MDA-MB-231	Triple-negative breast cancer cell line model
MIC	Minimum inhibitory concentration
Mia PaCa-2	Human pancreatic cancer cell line model
MMP	Matrix Metalloproteinase
mTOR	Mammalian Target of Rapamycin
NCI-H460	Large cell lung carcinoma cell line model
NF-κB	Nuclear Factor kappa-light-chain-enhancer of activated B cells
OA	Oleic acid
OVCAR-3	Ovarian carcinoma cell line model
PA	Palmitic acid
PANC-1	Human pancreatic epithelioid carcinoma cell line model
PARP	Poly (ADP-ribose) polymerase
PC-3	Prostate cancer cell line model
PGE2	Prostaglandin E2
PICO	Population; intervention; comparator; outcome
PI3K	Phosphoinositide 3-kinase
PLA2	Phospholipase A2
POA	Palmitoleic acid
PK	Mitogen-Activated Protein Kinase
ROO^•^	Peroxyl free organic radical
ROS	Reactive oxygen species
SA	Stearic acid
SKOV3	Ovarian adenocarcinoma cell line model
SNA	*Sambucus nigra* Agglutinin (Lectin)
STAT	Signal Transducer and Activator of Transcription
STAT3	Signal Transducer and Activator of Transcription 3
T47D	Breast ductal epithelial tumor cell line model
TBARS	Thiobarbituric acid reactive substances
TEK	Traditional ecological knowledge
TLR4	Toll-Like Receptor 4
TNF-α	Tumor necrosis factor alpha
TPA	12-O-tetradecanoylphorbol-13-acetate
TRP	Transient Receptor Potential
TRPA1	Transient Receptor Potential Ankyrin 1
TRPV1	Transient Receptor Potential Vanilloid 1
U-87	Human glioblastoma cell line model
UVB	Ultraviolet B
VEGF	Vascular Endothelial Growth Factor
WEFs	Wild Edible Fruits
